# Five solar cell parameters automatic extraction, within the one diode-solar cell model, using the implemented Simpson order 5 integration method, in an executable program

**DOI:** 10.1371/journal.pone.0346051

**Published:** 2026-04-22

**Authors:** Victor Tapio Rangel Kuoppa

**Affiliations:** Department of Physics, Lancaster University, Lancaster, United Kingdom; Sri Vidya Mandir Arts & Science College (Autonomous), INDIA

## Abstract

The Simpson order 5 integration method has been implemented in an executable program, to extract the five solar cell parameters, within the one-diode solar cell model. This integration method is used to integrate the Current minus the Short-Circuit Current, yielding a more accurate Co-Content function than the using the trapezoidal integration method. The program then fits the Co-Content function to a second-degree polynomial in two variables, namely, the Voltage and the Current minus the Short-Circuit Current, yielding six fitting constants, and the five solar cells are extracted from them. The program also calculates the standard deviations of the fitting constants, and from the, the standard deviations of the five solar cell parameters are also extracted. The results are given to the user in three text files, from where the user can easily access them or export them to other softwares. A video is also given, explaining how to use the executable program. The executable program provides the results in four seconds or less, in striking contrast with the tenths of minutes required by other methods, as they have to be implemented manually.

## Introduction

Humanity power demand will rise to 30 TW by year 2050 [1,2]. At the same time, mankind is well aware of the climate change, which has become so dramatic, that it has been started to be called climate catastrophe [3–6]. A possible solution to both problems is solar energy, as it is a larger source of free, eco-friendly energy [2].

The one-diode solar cell model is the one most used to investigated photovoltaic devices, due to its simplicity [7]. It consists of the series resistance (*R*_*s*_), the shunt resistance (*R*_*sh*_), the ideality factor (*n*), the saturation current (*I*_*sat*_), and the light current (*I*_*lig*_), which is also called photocurrent. The electric circuit of the one-diode model, and its Current-Voltage (*IV*) equation are commented in the following section. These five parameters provide important information for researchers investigating their photovoltaic devices. *I*_*sat*_ is related to Auger, Schockley-Read-Hall, or any surface recombination mechanisms [8], whilst *I*_*lig*_ depends on acceptor and donor life time and densities [9]. *n* provides information about transport mechanisms: in case *n* equals 1, it is minority carrier diffusion which is happening, while a value of 2 is evidence that recombination and/or generation of charge carriers inside the depletion region is occurring [10]. *R*_*s*_ is an indicator of the ohmic contact quality, whilst *R*_*sh*_, is indicated the crystal quality [11,12]. More information can be found in the literature [8–13].

The *I_V* equation (see Eq 1) cannot be solved for *V* or *I*, complicating the extraction of the five solar cell parameters. Several techniques have been suggested to deduce them. Some require some assumptions on one or more of the five solar cell parameters [14–21], whilst other are dependent on different radiation and/or maximum power measurements [14,15,19,22–27].

Other techniques consist in simulations and/or calculations, such as exponential model, Monte Carlo simulations, non-linear least-squares method, ab initio calculations, or artificial neuronal networks [28–37]. They do not extract directly the five solar cell parameters from the *I_V* data curves. Anyhow, a summary of their limitations can be read in [38]. Also recently, machine learning and artificial intelligence have been implemented to extract the solar cell parameters [39–43].

It is worth mentioning, before going into the one-diode solar cell model, that it is used to model second-generation solar cells. Nevertheless, it is a drift-diffusion model that is used to model third generation solar cells, such as perovskites-, and kesterites-based solar cells [44–50]. Further information about the drift-diffusion model can be read in the literature [44–50].

Only a handful of techniques are available to extract directly the five solar cell parameters from an *I_V* curve, independently of any illumination condition or doing any assumption [7,51–54]. In [7,52], two techniques were proposed to extract the five solar cell parameters, and they were later included into two iterative cycles [51,53]. In these iterative cycles, it was shown, that Cheung method, originally proposed to study Schottky contacts [55–60], was appliable also to solar cell *I_V* curves. In 2006, Ortiz-Conde *et al.* [54] used the Lambert function to obtain an expression of the *I_V* curve of solar cells. Then, they proposed the Co-Content function $CC(V,I)=\int_{\text{0}}^{V}\left(I-I_{sc}\right)dV$, ($I_{sc}=I(V=\text{0})$ is the short-circuit current) [54], showing that the five solar cell parameters can be extracted from the regression constants of CC(V,I) to ${C_{V\text{0}}+C}_{V\text{1}}V+C_{V\text{2}}V^{\text{2}}+C_{I\text{1}}\left(I-I_{sc}\right)+C_{I\text{2}}{\left(I-I_{sc}\right)}^{\text{2}}+C_{I\text{1}V\text{1}}V\left(I-I_{sc}\right)$ [54]. It is worth mentioning that other techniques are used when the solar cell model includes two or more parallel diodes. These multi-diode solar cell models are not the intention of this article, but the reader can find more about them in [ 61–68]. An excellent review about the extraction techniques on these alternative solar cell models can be found in [69].

The application of the Ortiz-Conde *et al.* method [54] to noiseless *I_V* curves, with a percentage noise $p_{n}=\text{0}\%$, and a density of measured points per voltage $P_{V}=\text{1}\text{0}\text{0}\text{0}\frac{measuredpoints}{V}$ (see Section 3 in [7]), extracted excellently the five solar cell parameters [7]. However, when it was applied into real CdTe- and CIGS-based solar cell measured *I_V* curves, unrealistic solar cell parameter extraction occurred, due to the pernicious effect of noise (see Section 4 in [52]). This reveals that the accurate calculation of CC(V,I) is fundamental, depending strongly on$P_{V}$ and $p_{n}$. Studies have revealed that the pernicious effect of the noise can be reduced, in some cases, increasing the value of $P_{V}$ [70–73]. In those studies [70–73], CC(V,I) was quantified using the trapezoidal integration tecnique, and an increase of $P_{V}$ reduced the deleterious effect of the noise, in some cases, providing a more accurate CC(V,I), and then, the solar cell parameter extraction was more accurate [70–73]. However, values of $P_{V}$ as large as 50,001 $\frac{measuredpoints}{V}$ are necessary, causing the computation time and data management to increase largely. Alternative solutions appeared: the Newton-Cotes quadrature integration, the 3/8 rule integration, and Simpson integration methods were implemented, to minimize the pernicious effect of noise [74–76], while other studies, the CC(V,I) was computed, integrating a polynomial fit of $I-I_{sc}$ [77,78]. All these described methodologies were successful on reducing the effect of noise, nevertheless, software like Labview or Origin are needed to implement them, causing researchers to purchase these software licenses, and to use worthy time implementing them. Hence, it would be very convenient for the solar energy devices research community, to have an executable program that could automatically, quick and accurately extract the five solar cell parameters and their standard deviations, (considered in this article as the standard errors), together with any matrixes and vectors involved in the calculations, from any *I_V* curve.

This brief discussion explains the reason of this research: to make available to the solar energy photovoltaic research community an automatic executable program, that deduces the five solar cell parameters and any other valuable information of any *I_V* curve, based on the Ortiz-Conde *et al.* method [54].

This study is structured as follows. After this section 1, the section 2 follows, where the one-diode solar cell model is briefly commented. Also, a summary of the Ortiz-Conde *et al.* method [54] is given. In section 3, the polynomial regression of CC(V,I) to ${C_{V\text{0}}+C}_{V\text{1}}V+C_{V\text{2}}V^{\text{2}}{+C}_{I\text{1}}\left(I-I_{SC}\right)+C_{I\text{2}}{\left(I-I_{SC}\right)}^{\text{2}}+C_{I\text{1}V\text{1}}V\left(I-I_{SC}\right)$ is analysed, including the standard deviations (the standard errors) computation. Also in section 3, a flow diagram of the program is exposed, together with a numerical example and a Video in the Supplementary Material. Results are then commented in section 4. Finally, conclusions are given in section 5.

## Summary of the Ortiz-conde *et al.* method

**Fig 1:**
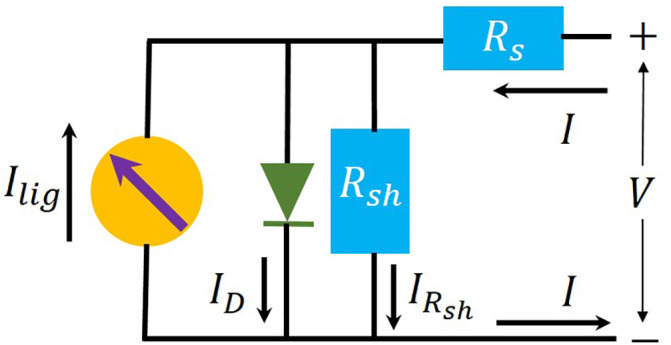
Electric circuit of the one-diode solar cell model. The ${𝐈}_{lig}$ is the light current, generated by the photons, ${𝐈}_{D}$ is the current through the diode, ${𝐈}_{R_{sh}}$ is the current through the shunt resistance, I is the current coming and going out the solar cell, and 𝐕 is the applied voltage applied on the solar cell.


For the sake of completeness, first, the one-diode solar cell model circuit and the *I_V* equation of the one-diode solar cell model are shown in Fig 1 and in Eq. (1) [7]. A summary of the Ortiz-Conde *et al.* method [54] comes next.

$$I=I_{sat}\left(exp\left(\frac{V-IR_{s}}{nkT}\right)-\text{1}\right)+\frac{V-IR_{s}}{R_{sh}}-I_{lig}$$ (1)

Ortiz-Conde *et al.* proposed, in 2006, the Co-Content CC(V,I) function as [54]


$$CC(V,I)=\int_{\text{0}}^{V}\left(I-I_{sc}\right)dV,$$ (2)

where $I_{sc}=I(V=\text{0})$ is the short-circuit current.

In their study, they showed that, after doing polynomial regression of Eq 2 to the following second-degree polynomial in V and $I-I_{SC}$:


$$C_{V\text{0}}+C_{V\text{1}}V+C_{V\text{2}}V^{\text{2}}+C_{I\text{1}}\left(I-I_{SC}\right){+C}_{I\text{2}}{\left(I-I_{SC}\right)}^{\text{2}}+C_{I\text{1}V\text{1}}V\left(I-I_{SC}\right),$$ (3)

*R*_*s*_, *R*_*sh*_, *n*, and *I*_*lig*_ parameters could be extraccted from the regression constants $C_{V\text{1}}$, $C_{I\text{1}}$, $C_{V\text{2}}$, and $C_{I\text{2}}$ using [54]


$$R_{sh}=\frac{\text{1}}{\text{2}C_{V\text{2}}},$$ (4)

$$R_{s}=\frac{A-\text{1}}{\text{4}C_{V\text{2}}},$$ (5)

$$n=\frac{C_{V\text{1}}(A-\text{1})+\text{4}C_{I\text{1}}C_{V\text{2}}}{\text{4}V_{th}C_{V\text{2}}},$$ (6)

$$I_{lig}=-\frac{(\text{1}+A)\left(C_{V\text{1}}+I_{sc}\right)}{\text{2}}-\text{2}C_{I\text{1}}C_{V\text{2}},$$ (7)

where $V_{th}=kT$ is the thermal velocity, whilst T the absolute temperature, k the Boltzmann constant, and $A=\sqrt{\text{1}+\text{1}\text{6}C_{I\text{2}}C_{V\text{2}}}$.

*I*_*sat*_ is then computed using


$$I_{sat}=B\left(I+I_{lig}-\frac{V-IR_{s}}{R_{sh}}\right),$$ (8)

evaluating it the largest possible V (see Section 4 in [7] for further details).

The function B is $B={\left(exp((V-IR_{s})/nkT)-\text{1}\right)}^{-\text{1}}$.

The expressions of the first order errors of *R*_*s*_, *R*_*sh*_, *n*, and *I*_*lig*_, i.e., $\Delta R_{s}$, $\Delta R_{sh}$, Δn, and $\Delta I_{lig}$, were deduced in Section 3.2 in [52], while the expression for the first error of *I*_*sat*_, i.e., $\Delta I_{sat}$, was deduced in Section 2 in [56], as function of the standard deviations of $C_{V\text{0}}$, $C_{V\text{1}}$, $C_{V\text{2}}$, $C_{I\text{1}}$, $C_{I\text{2}}$, $C_{V\text{1}I\text{1}}$, i.e., $\Delta C_{V\text{0}}$, ${\Delta C}_{V\text{1}}$, ${\Delta C}_{V\text{2}}$, ${\Delta C}_{I\text{1}}$, $\Delta C_{I\text{2}}$, and $\Delta C_{V\text{1}I\text{1}}$, and they are:


$${\Delta R}_{sh}=-\frac{\Delta C_{V\text{2}}}{\text{2}\overset{\text{2}}{\underset{V\text{2}}{C}}},$$ (9)

$$\Delta R_{s}=\frac{\text{1}}{\text{2}}\left\{\left(\frac{A-\left(\text{1}+\text{8}C_{V\text{2}}C_{I\text{2}}\right)}{A\overset{\text{2}}{\underset{V\text{2}}{C}}}\right)\Delta C_{V\text{2}}+\frac{\text{8}\Delta C_{I\text{2}}}{A}\right\},$$ (10)

$$\Delta n=\left(\frac{A-\text{1}}{\text{4}V_{th}C_{V\text{2}}}\right)\Delta C_{V\text{1}}+\frac{C_{V\text{1}}\left(A-\left(\text{1}+\text{8}C_{V\text{2}}C_{I\text{2}}\right)\right)}{\text{4}V_{th}AC_{V\text{2}}}\Delta C_{V\text{2}}+\frac{\text{1}}{V_{th}}\Delta C_{I\text{1}}+\frac{\text{2}C_{V\text{1}}}{AV_{th}}\Delta C_{I\text{2}},$$ (11)

$$\Delta I_{lig}=-\left(\frac{\text{1}+A}{\text{2}}\right)\Delta C_{V\text{1}}-\left\{\frac{\text{4}C_{I\text{2}}\left(C_{V\text{1}}+I_{sc}\right)}{A}+\text{2}C_{I\text{1}}\right\}\Delta C_{V\text{2}}-\text{2}C_{V\text{2}}\Delta C_{I\text{1}}-\frac{\text{4}\left(C_{V\text{1}}+I_{sc}\right)C_{V\text{2}}}{A}\Delta C_{I\text{2}}$$ (12)

$$\begin{array}{cc} \Delta I_{sat}= & B\left\{\text{1}+\frac{R_{s}}{R_{sh}}-\left(\frac{R_{s}}{nkT}\right)\left\{B+\text{1}\right\}\left\{I+I_{lig}-\frac{V-IR_{s}}{R_{sh}}\right\}\right\}\Delta I+B\Delta I_{lig}\\ & +BI\left\{\frac{\text{1}}{R_{s}}-\left(\frac{B+\text{1}}{nkT}\right)\left(I+I_{lig}-\frac{V-IR_{s}}{R_{sh}}\right)\right\}\Delta R_{s}+B\left(\frac{V-IR_{s}}{{R_{sh}}^{\text{2}}}\right)\Delta R_{sh}-B(B+\text{1})\\ & \left(\frac{IR_{s}}{n^{\text{2}}kT}\right)\left(I+I_{lig}-\frac{V-IR_{s}}{R_{sh}}\right)\Delta n, \end{array}$$ (13)

where ΔI is the amperemeter precision.

The implementation of the Ortiz-Conde *et al.* method [54] is discussed in the following section.

## *I_V* Simulation and implementation of the Ortiz-conde *et. al* method

The *I_V* curves were simulated using the C program used in [7]. The solar cell parameters used in the simualtions were were n=2.5, *R*_*sh*_ = 1 kΩ, *R*_*s*_ = 1 Ω, *I*_*lig*_ = 1 mA, and *I*_*sat*_ = 1µA, in the [0 V, 1 V] voltage range. *R*_*sh*_, *R*_*s*_, *I*_*sat*_, and *I*_*lig*_ are powers of 10, to simplify the comparison with the extracted solar cell parameters by the program. Also, second generation laboratory-made solar cells have this relation among these parameters: *R*_*s*_ and *I*_*sat*_ are three orders of magnitude smaller than *R*_*sh*_ and *I*_*lig*_, respectively [79–98]. The *I_V* curves were first simulated noiseless, *i.e.,* $p_{n}=\text{0}\%$, and then, a second group of *I_V* curves was simulated, adding a $p_{n}=\text{0}.\text{01}\%$, 0.05%, and 0.1% additional noise, to test the precision of the program in *I_V* curves. They are labelled as the $p_{n}=\text{0}\%$, adding the text “CpercnoiseI”, where C is the percentage noise.

Let’s assume we have an *I_V* data set of *N* data pairs, i.e., $\left\{\left(V_{i},I_{i}\right)withi=\text{0},\text{1},\ldots ,N-\text{1}\right\}$. In this study, the first data point is indexed as zero, meaning that the last data point is the *N* – 1 point. The pseudo-code of the program can be found in the Supplementary Material.

The first *I_V* data point is $\left(V_{\text{0}}=\text{0}V,I_{\text{0}}=I_{sc}\right)$, and the program calculates $I_{sc}$ as this first data point, and also the program calculates the voltage step, i.e., ΔV, as, $\Delta V=V_{\text{1}}-V_{\text{0}}$. This explains why one of the conditions of the *I_V* data text file is that all the voltage points must be equally spaced (see text below), as the program assumes it to be constant value through the whole program. For the next step, the program calculates $I-I_{sc}$ as ${\left(I-I_{sc}\right)}_{i}=I_{i}-I_{sc}$.

The program then computes CC(V,I), using the trapezoidal integration formula for the ${CC}_{\text{1}}$, the Newton-Cotes quadrature integration formula for the ${CC}_{\text{2}}$, the 3/8 integration rule for the ${CC}_{\text{3}}$, and the Boole’s integration rule for the ${CC}_{\text{4}}$. The subsequent ${CC}_{i}$ are calculated using the Simpson Order 5 integration method [99]


$${CC}_{\text{0}}=\text{0}$$ (14)

$${CC}_{\text{1}}={CC}_{\text{0}}+\left(\frac{{\left(I-I_{sc}\right)}_{\text{1}}+{\left(I-I_{sc}\right)}_{\text{0}}}{\text{2}}\right)\Delta V$$ (15)

$${CC}_{\text{2}}={CC}_{\text{0}}+\frac{\Delta V}{\text{3}}\left({\left(I-I_{sc}\right)}_{\text{2}}+\text{4}{\left(I-I_{sc}\right)}_{\text{1}}+{\left(I-I_{sc}\right)}_{\text{0}}\right)$$ (16)

$${CC}_{\text{3}}={CC}_{\text{0}}+\frac{\text{3}\Delta V}{\text{8}}\left({\left(I-I_{sc}\right)}_{\text{3}}+\text{3}{\left(I-I_{sc}\right)}_{\text{2}}+\text{3}{\left(I-I_{sc}\right)}_{\text{1}}+{\left(I-I_{sc}\right)}_{\text{0}}\right)$$ (17)

$${CC}_{\text{4}}={CC}_{\text{0}}+\frac{\text{2}\Delta V}{\text{4}\text{5}}\left(\text{7}{\left(I-I_{sc}\right)}_{\text{4}}+\text{3}\text{2}{\left(I-I_{sc}\right)}_{\text{3}}+\text{1}\text{2}{\left(I-I_{sc}\right)}_{\text{2}}+{\text{3}\text{2}\left(I-I_{sc}\right)}_{\text{1}}+\text{7}{\left(I-I_{sc}\right)}_{\text{0}}\right)$$ (18)

$$\begin{array}{cc} {CC}_{i}= & {CC}_{i-\text{5}}+\frac{\text{5}\Delta V}{\text{2}\text{8}\text{8}}(\text{1}\text{9}{\left(I-I_{sc}\right)}_{i}+\text{7}\text{5}{\left(I-I_{sc}\right)}_{i-\text{1}}+\text{5}\text{0}{\left(I-I_{sc}\right)}_{i-\text{2}}+{\text{5}\text{0}\left(I-I_{sc}\right)}_{i-\text{3}}\\ & +\text{7}\text{5}{\left(I-I_{sc}\right)}_{i-\text{4}}+\text{1}\text{9}{\left(I-I_{sc}\right)}_{i-\text{5}})fori=\text{5},\ldots ,N-\text{1} \end{array}$$ (19)

An example of the implementation of the method is given in the Supplementary Material, where the analysis of the file “I-V9points.txt” is exposed. Also, the statistical procedure is explained on how to calculate the standard deviations, the diagram flow, the program steps, the conditions the *I_V* text file data should have to be properly read by the program, and a numerical table with the results is given.

These results exposed in the Supplementary Material are discussed in the next Section.

### Ethics statements

No experiments with animals or humans were done.

## Results and discussion

**Fig 2:**
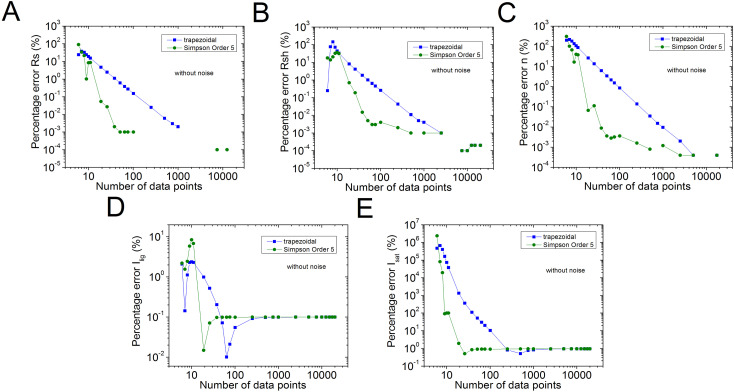
Graph (in green) of the results reported in Table 2 (no noise case), for percentage errors of (a) ${𝐑}_{s}$, (b) ${𝐑}_{sh}$, (c) *n*, (d) *I*_*lig*_, and (e) *I*_*sat*_, relative to the original of 𝐧=2.5, *R*_*s*_ = 1 Ω, *R*_*sh*_= 1 kΩ, *I*_*sat*_ = 1µA, and ${𝐈}_{lig}$ = 1 mA parameters. In blue colour, are the results obtained using the trapezoidal integration provided by the software Origin.

**Fig 3:**
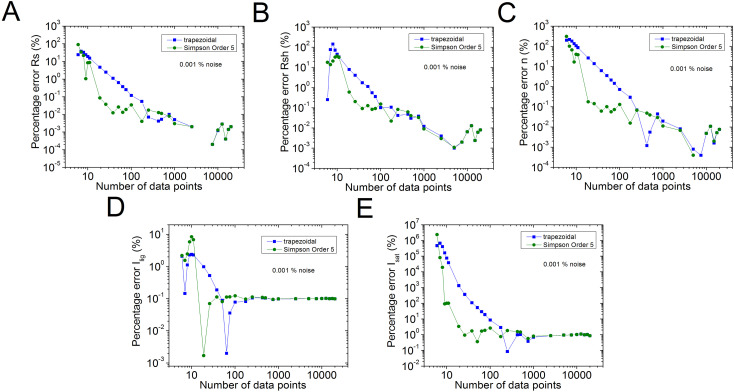
Graph (in green) of the results reported for the ${𝐩}_{n}=0.001\%$ of noise, for percentage errors of (a) (a) ${𝐑}_{s}$, (b) ${𝐑}_{sh}$, (c) *n*, (d)*I*_*lig*_, and (e) *I*_*sat*_, relative to the original of 𝐧=2.5, *R*_*s*_ = 1 Ω, *R*_*sh*_ = 1 kΩ, *I*_*sat*_ = 1µA, and ${𝐈}_{lig}$ = 1 mA parameters, obtained using the program CCSimpsonOrder5.exe, reported in this article. In blue colour, are the results obtained using the trapezoidal integration provided by the software Origin.

**Fig 4:**
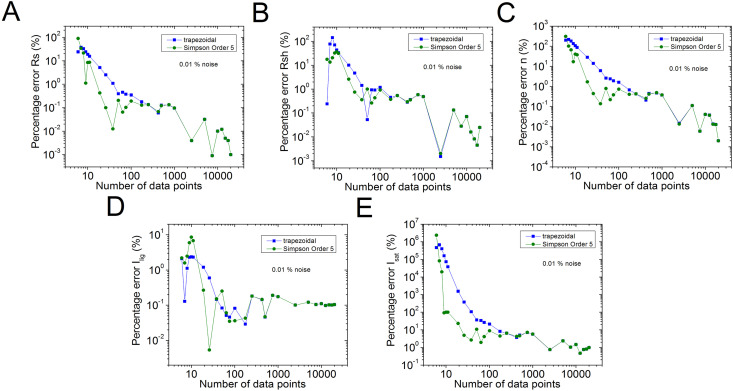
Graph (in green) of the results reported for the ${𝐩}_{n}=$ 0.01% of noise, for percentage errors of (a) ${𝐑}_{s}$, (b) ${𝐑}_{sh}$, (c) n, (d) Ilig, and (e) Isat, relative to the original of 𝐧=2.5, Rs = 1 Ω, Rsh = 1 kΩ, Isat = 1µA, and ${𝐈}_{lig}$ = 1 mA parameters, obtained using the program CCSimpsonOrder5.exe, reported in this article. In blue colour, are the results obtained using the trapezoidal integration provided by the software Origin.

**Fig 5:**
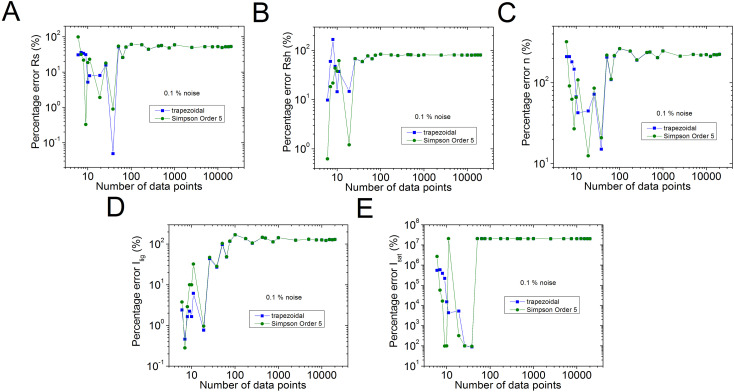
Graph (in green) of the results reported for the ${𝐩}_{n}=$ 0.1% of noise, for percentage errors of (a) ${𝐑}_{s}$, (b) ${𝐑}_{sh}$, (c) n, (d) Ilig, and (e) Isat, relative to the original of 𝐧=2.5, Rs = 1 Ω, Rsh = 1 kΩ, Isat = 1µA, and ${𝐈}_{lig}$ = 1 mA parameters, obtained using the program CCSimpsonOrder5.exe, reported in this article. In blue colour, are the results obtained using the trapezoidal integration provided by the software Origin.

The percentage errors, relative to the original values of n=2.5, *R*_*s*_ = 1 Ω, *R*_*sh*_ = 1 kΩ, *I*_*sat*_ = 1µA, and $I_{ph}$ = 1 mA, of the results reported in S2 Table in the Supplementary Material S1 File are plot in Fig 2 in blue colour. Also, for comparison purposes, with black colour, the percentage errors using the trapezoidal integration method provided by the software Origin, are shown. In  Figs 3–5, the percentage errors for the cases of $p_{n}=\text{0}.\text{001}\%$, 0.01%, and 0.1% of noise are exposed, respectively.

As can be seen from S2 Table and Fig 2, excellent parameter extraction was achieved using the order 5 Simpson integration, in the no noise case, obtaining the five solar cell parameters with less than 1% error, with just N≥26, while N≥251 should be used, in case the trapezoidal integration method is applied. Something similar happens in the case of $p_{n}=$0.01%: in case the order 5 Simpson integration is used, the five solar cell parameters can be obtained with 1% error or less, with N≥76, however N≥751, in case the trapezoidal integration method is used.

When $p_{n}\geq$0.1%, no evident advantage of the order 5 Simpson integration over the trapezoidal integration is observed, and $R_{s}$, $R_{sh}$, *n*, and *I*_*lig*_ can be deduced with 1%, 10%, 7%, and 10% percentage errors, using N≥1001, when $p_{n}=$0.1%, and in case $p_{n}=$1%, the percentage errors for these four solar cell parameters are around 7%, 10%, 20%, and 40%, also for N≥1001, respectively. In the case of *I*_*sat*_, if $p_{n}=$0.1%, it is not possible to obtain it with less than 50%, and if case $p_{n}=$1%, it stays around 200% error, even if N=20001.

It is being investigated if other integration methods could improve the results exposed in this article, such as Monte Carlo integration, Gauss quadrature, Simpson integration of order 6, including ab initio calculations. They will be reported elsewhere.

The user should notice that these results are valid for the one-diode solar cell model. They are not necessarily valid for other solar cell models, such as two-, three-, or multi-diode solar cell models, or models including capacitors.

## Validation on experimental *I_V* curves

**Fig 6:**
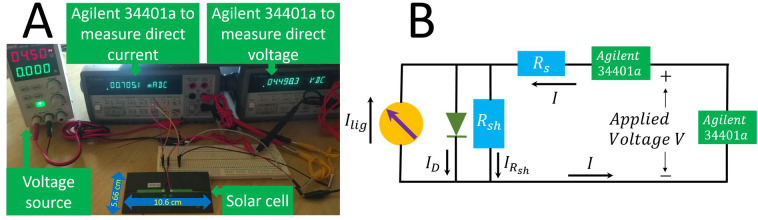
A Electrical setup, to record the *I_V* curves of a solar cell, and B electrical circuit of the setup given in A.

The program was validated on *I_V* curves measured on a commercial solar cell. The setup can be seen in Fig 6A. where two Agilent 34401a multimeters were used to, one in series to measured the direct current, and the other in parallel with the voltage source, to confirm and record the applied voltage, as can be seen in the circuit given in Fig 6B.

The *I_V* curves were measured in darkness and under illumination, and they are exposed in Fig 6A for the darkness case, and Fig 6B, for the illumination case. For each value of *V*, the *I* was measured 1, 10, 100, and 1000 times, and averaged, to diminish the noise presence.

**Fig 7:**
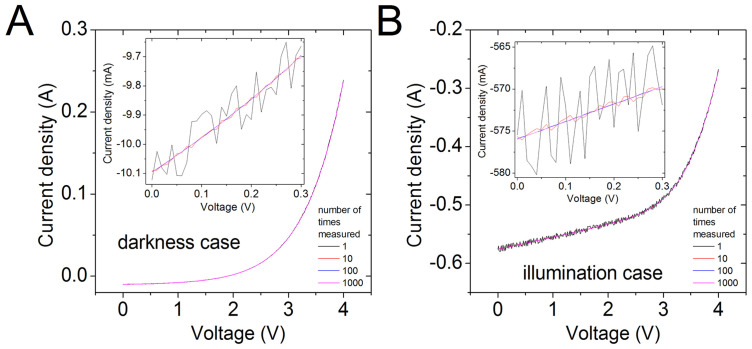
A *I_V* curves measured in darkness and B under illumination. **For each value of *V*, the *I* was measured 1 time (black curve), 10 times (red curve), 100 times (blue curve), and 1000 times (magenta curve), and averaged, to diminish the presence of noise.** In the insets, the [0 V, 0.3 V] range is shown, to allow the reader a clearer view of the presence of noise.

**Fig 8:**
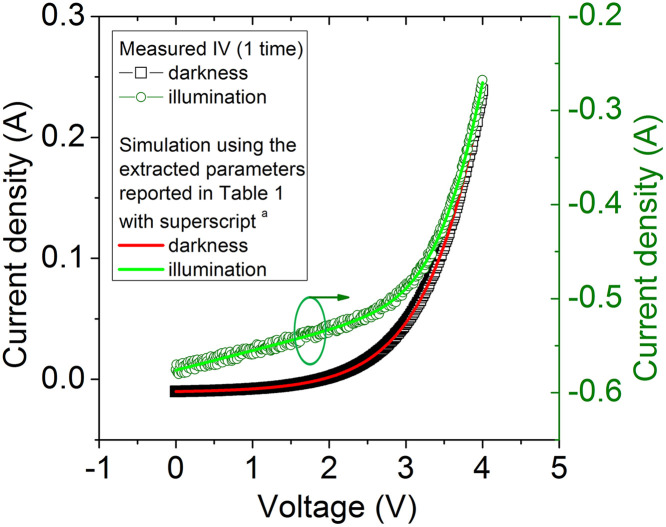
Simulations done with the extracted parameters, using CycleB proposed in [46,48] and reported with superscript ^a^ in Table 1, on the 1 time measured (black curves), and reported as red curve for the darkness case, and green curve for the illumination case. The simulations reasonably reproduced the measured *I_V* curves, confirming good parameters extraction.

The CycleB, proposed in [51,53], was used in the *I_V* curves measured 1 time, exposed in Fig 7 (black lines), to extract the solar cell parameters, and they are reported in Table 1 with the superscript ^a^. Simulations done with these extracted parameters were done and are shown as red and green lines in Fig 7, and they reasonably reproduce the *I_V* measurements done 1 time (black lines in Fig 7) confirming good parameter deduction Fig 8.

**Table 1:** ^a^ using CycleB, ^b^ using trapezoidal, ^c^ using program CCSimpsonOrder5.exe.

In darkness					
Number of times measured and averaged	Rs ± ΔRs (ohms)	Rsh ± ΔRsh (ohms)	n ± Δn	Ilig ± ΔIlig (A)	IsatMaxV ± ΔIsatMaxV (A)
1	^a^ 0.424 ± 0.08 ^b^ −0.5 ± 0.2 ^c^ −0.2 ± 0.1	^a^ 2703 ± 15 ^b^ 124 ± 45 ^c^ 443 ± 42	^a^ 24 ± 0.2 ^b^ 1 ± 2 ^c^ 3 ± 2	^a^ 0.01 ^b^ 0.001 ± 0.0002 ^c^ 0.011 ± 0.0002	^a^ 4.59e-4 ± 7e-6 ^b^ 3e-25 ± 1e-22 ^c^ 8e-23 ± 4e-23
10	^b^ −0.5 ± 0.03 ^c^ 0.4 ± 0.02	^b^ 517 ± 235 ^c^ 2320 ± 145	^b^ 11.2 ± 2.3 ^c^ 23.2 ± 0.3	^b^ 0.00554 ± 3e-4 ^c^ 0.01054 ± 3e-5	^b^ 0.0044 ± 1e-4 ^c^ 0.01054 ± 3e-5
100	^b^ 0.1 ± 0.03 ^c^ 0.423 ± 0.002	^b^ 1321 ± 59 ^c^ 2691 ± 19	^b^ 13 ± 1.3 ^c^ 24 ± 0.03	^b^ 0.007559 ± 3e-5 ^c^ 0.010559 ± 3e-6	^b^ 0.00418 ± 1e-4 ^c^ 0.000418 ± 1e-6
1000	^b^ 0.3242 ± 0.0002 ^c^ 0.4242 ± 0.0002	^b^ 2011 ± 5 ^c^ 2706 ± 2	^b^ 15 ± 1 ^c^ 24.036 ± 0.003	^b^ 0.00956 ± 2e-7 ^c^ 0.01056 ± 3e-7	^b^ 0.000922 ± 4e-7 ^c^ 0.000422 ± 2e-7
Under illumination					
1	^a^ 1.1 ± 0.3 ^b^ −0.6 ± 0.1 ^c^ 0.3 ± 0.1	^a^ 47 ± 2 ^b^ 11 ± 3 ^c^ 62 ± 1	^a^ 13 ± 1 ^b^ −11.5 ± 2.1 ^c^ −6.5 ± 0.6	^a^ 0.589 ^b^ 2.17899 ± 4e-4 ^c^ 0.57899 ± 7e-5	^a^ 1e-6 ± 2e-7 ^b^ 3 ± 1 ^c^ 0.91 ± 0.09
10	^b^ 0.11 ± 0.2 ^c^ 0.81 ± 0.05	^b^ 17.1 ± 2 ^c^ 51.4 ± 0.5	^b^ 2.2 ± 0.6 ^c^ 7.2 ± 0.6	^b^ 1.3355 ± 0.003 ^c^ 0.5855 ± 0.0003	^b^ -7e-8 ± 3e-9 ^c^ 1e-10 ± 3e-12
100	^b^ 0.461 ± 0.009 ^c^ 1.061 ± 0.007	^b^ 21.03 ± 0.7 ^c^ 47.03 ± 0.05	^b^ 5.37 ± 0.39 ^c^ 13.37 ± 0.09	^b^ 0.9796 ± 4e-6 ^c^ 0.58896 ± 4e-5	^b^ −2.507e-4 ± 2e-9 ^c^ 3.802e-6 ± 2e-9
1000	^b^ 0.8964 ± 0.0009 ^c^ 1.0643 ± 0.0007	^b^ 36.13 ± 0.01 ^c^ 46.98 ± 0.005	^b^ 9.357 ± 0.02 ^c^ 13.445 ± 0.008	^b^ 0.489 ± 2e-5 ^c^ 0.589 ± 4e-6	^b^ −3.0409e-6 ± 2e-9 ^c^ 2e-6 ± 2e-10

As can be seen from Table 1, the trapezoidal integration does not yield correct parameter extraction, compared with those extracted using CycleB, and shown with the superscript ^a^, but it nevertheless it improves as the number of averaged data increase from only one, to one thousand. In contrast, the program CCSimpsonOrder5.exe already provide reasonably the same parameters as those extracted using CycleB, once the number of averaged data is one hundred or one thousand. It is worth mentioning, that the program CCSimpsonOrder5.exe provides the results in less than four seconds, in striking contrast with the tenths of minutes required using CycleB or the trapezoidal integration, as they have to be implemented manually.

## Conclusion

In this article, a C++ - based executable program has been reported and made available to the photovoltaic community, where an algorithm based in the order 5 Simpson integration formula has been implemented, to compute more accurately the Co-Content function, to obtain the five solar cell parameters, together with their standard errors (the standard deviations). It has been tested in ideal (noiseless) *I_V* curves, and *I_V* curves with $p_{n}=$ 0.01% noise, and excellent parameter extraction is obtained, with less than 1% error, with just N≥26, and N≥76, respectively. The program yields to the user all the information in a three text files, to allow the user an easy application of them.

The program was implemented on *I_V* curves measured in a commercial solar cell. The program CCSimpsonOrder5.exe extracts correctly the five solar cell parameters, provided that the *I_V* curves were measured averaging one hundred or one thousand points per each voltage measured, in order to reduce the presence of noise. In case of only ten averages, or only one single measurement per voltage, the parameter extraction is not that accurate. This reveals the pernicious effect of noise, and the importance to do at least one hundred averaged measurements per each voltage measured, to decrease the pernicious effect of noise, to allow the program CCSimpsonOrder5.exe to have accurate enough *I_V* data, to correctly extract the solar cell parameters.

It is being investigated if Simpson integration of 6 order, Monte Carlo integration, ab initio calculations or Gauss quadrature, could provide better results than those reported in this study. Also, it is expected that the application of program in computers with more powerful and moder processors, would shorten the computational times.

It is expected that the program provided in this study, will help the photovoltaic solar energy research community to enhance their photovoltaic device research.

## Supporting information

S1 File Supp Material Revision 1 Article PloS One CC SimpsonOrder5.docx. Supplementary Material of the article Full title: Five solar cell parameters automatic extraction, within the one diode-solar cell model, using the implemented Simpson order 5 integration method, in an executable program. Short title: Automatic solar cell parameters extraction, using Simpson order 5 integration method, in an executable program.
(DOCX)

S2 File Pseudocode CCSimpsonOrder5.docx. Pseudo-code of program CCSimpsonOrder5.exe.
(DOCX)

S1 FigSchematic diagram, on the program steps done to obtain ${𝐑}_{𝐬}+\Delta {𝐑}_{𝐬},{𝐑}_{𝐬𝐡}+\Delta {𝐑}_{𝐬𝐡},𝐧+\Delta 𝐧,{𝐈}_{𝐥𝐢𝐠}+\Delta {𝐈}_{𝐥𝐢𝐠},𝐚𝐧𝐝{𝐈}_{𝐬𝐚𝐭}+\Delta {𝐈}_{𝐬𝐚𝐭}$.(BMP)

S2 FigVisual explanation, on how the 𝐂𝐂(𝐕,𝐈) is calculated in S1 Table, in A using the trapezoidal integration, in B the Newton Cotes integration, in C, the 3/8 integration, in D the Boole’s integration, in E the order 5 Simpson integration, F the order 5 Simpson integration, in F the order 5 Simpson integration, adding it to the integration obtained in A, in G the order 5 Simpson integration, adding it to the integration obtained in B, and in H the order 5 Simpson integration, adding it to the integration obtained in C.(TIF)

S1 Table Example on the application of program CCSimpsonOrder5.exe, on IV data file I-V9points.txt, available in the Supplementary Material. First, in Column 1 and Column 2, namely Col1 and Col2, the IV data from file I-V9points.txt, is given. Afterwards, in Col3, I – Isc, is reported, as the value of Column 2, minus the value (Line 1, Column 2), i.e., Col2 – (L1, Col2). Next, in Col4, an explanation on how CC(V, I) was calculated is given, using the trapezoidal integration method (Eq. (15)) in L2, the Newton Cotes integration (Eq. (16)) method in L3, the 3/8 rule (Eq. (17)) in L4, and then, from L5 to L8, the Boole’s integration formula (Eq. (18)), adding it to value in the previous line and same column is shown. For the sake of clarity, the numerical calculations are given. Finally, in Col5 the final value of CC(V, I) is reported.
(DOCX)

S2 Table${𝐂}_{𝐕0}\pm {\Delta 𝐂}_{𝐕0},{𝐂}_{𝐕1}\pm {\Delta 𝐂}_{𝐕1},{{𝐂}_{𝐕2}\pm {\Delta 𝐂}_{𝐕2},𝐂}_{𝐈1}\pm {\Delta 𝐂}_{𝐈1},{𝐂}_{𝐈2}\pm \Delta {𝐂}_{𝐈2},{𝐂}_{𝐕1𝐈1}\pm {\Delta 𝐂}_{𝐕1𝐈1},{𝐑}_{𝐬}\pm \Delta {𝐑}_{𝐬},{𝐑}_{𝐬𝐡}\pm \Delta {𝐑}_{𝐬𝐡},𝐧\pm \Delta 𝐧,{𝐈}_{𝐥𝐢𝐠}\pm \Delta {𝐈}_{𝐥𝐢𝐠},𝐚𝐧𝐝{𝐈}_{𝐬𝐚𝐭}\pm \Delta {𝐈}_{𝐬𝐚𝐭}$ obtained using the program CCSimpsonOrder5.exe.The value “nan” means “not-a-number”, and it appears in N=6 case, as the standard deviation is undetermined, as explained in the text (see Eq. (35)).(DOCX)
