# Supplementary material for: Five solar cell parameters automatic extraction, within the one diode-solar cell model, using the implemented Simpson order 5 integration method, in an executable program
Source: PLoS One. 2026 Apr 22;21(4):e0346051. doi: 10.1371/journal.pone.0346051 (PMC13102238; doi:10.1371/journal.pone.0346051)
Supplement: S1 File — Supplementary Material of the article Full title: Five solar cell parameters automatic extraction, within the one diode-solar cell model, using the implemented Simpson order 5 integration method, in an executable program. Short title: Automatic solar cell parameters extraction, using Simpson order 5 integration method, in an executable program. (DOCX) [file pone.0346051.s001.docx]

***Supplementary Material of the article***

**Full title: Five solar cell parameters automatic extraction, within the one diode-solar cell model, using the implemented Simpson order 5 integration method, in an executable program.**

**Short title: Automatic solar cell parameters extraction, using Simpson order 5 integration method, in an executable program.**

The $\vec{CC}$ vector is defined as

$\vec{CC}=\left( \begin{matrix} {CC}_{0} \\ {CC}_{1} \\ \vdots\\ {CC}_{N-2} \\ {CC}_{N-1} \end{matrix} \right)$ (20)

To calculate the polynomial regression to $C_{V0}+C_{V1}V+C_{V2}V^{2}+C_{I1}\left( I-I_{sc} \right)+C_{I2}\left( I-I_{sc} \right)^{2}+C_{I1V1}V\left( I-I_{sc} \right)$ and obtain $C_{V0}$, $C_{V1}$, $C_{V2}$, $C_{I1}$, $C_{I2},$and $C_{I1V1}$, the following function is proposed

$$l\left( C_{V0}, C_{V1}, C_{V2}, C_{I1}, C_{I2}, C_{I1V1} \right)=$$

$\sum_{i=0}^{N-1} \left( C_{V0}+C_{V1}V_{i}+C_{V2}V_{i}^{2}+C_{I1}\left( I-I_{sc} \right)_{i}+C_{I2}\left( I-I_{sc} \right)_{i}^{2}+C_{I1V1}V\left( I-I_{sc} \right)-{CC}_{i} \right)^{2}$ (21)

The function $l\left( C_{V0}, C_{V1}, C_{V2}, C_{I1}, C_{I2}, C_{I1V1} \right)$ is minimized respect $C_{V0}$, $C_{V1}$, $C_{V2}$, $C_{I1}$, $C_{I2},$and $C_{I1V1}$, i.e., the following equations are calculated and solved: $\frac{\partial l}{\partial C_{V0}}= \frac{\partial l}{\partial C_{V1}}=\frac{\partial l}{\partial C_{V2}}= \frac{\partial l}{\partial C_{I1}}=\frac{\partial l}{\partial C_{I2}}= \frac{\partial l}{\partial C_{I1V1}}=$0, providing the following set of equations:

$$C_{V0}N +C_{V1}\sum V_{i}+C_{V2}\sum V_{i}^{2}+C_{I1}\sum\left( I-I_{sc} \right)_{i}+C_{I2}\sum\left( I-I_{sc} \right)_{i}^{2}+C_{I1V1}\sum V_{i}\left( I-I_{sc} \right)_{i}=\sum{CC}_{i} (22)$$

$$C_{V0}\sum V_{i} + C_{V1}\sum V_{i}^{2}+C_{V2}\sum V_{i}^{3}+C_{I1}\sum{V_{i}\left( I-I_{sc} \right)}_{i}+C_{I2}\sum V_{i}\left( I-I_{sc} \right)_{i}^{2}+C_{I1V1}\sum V_{i}^{2}\left( I-I_{sc} \right)_{i}=\sum V_{i}{CC}_{i} (23)$$

$$C_{V0}\sum V_{i}^{2}+ C_{V1}\sum V_{i}^{3}+C_{V2}\sum V_{i}^{4}+C_{I1}\sum{V_{i}^{2}\left( I-I_{sc} \right)}_{i}+C_{I2}\sum V_{i}^{2}\left( I-I_{sc} \right)_{i}^{2}+C_{I1V1}\sum V_{i}^{3}\left( I-I_{sc} \right)_{i}=\sum V_{i}^{2}{CC}_{i} (24)$$

$$C_{V0}\sum\left( I-I_{sc} \right)_{i} + C_{V1}\sum V_{i}\left( I-I_{sc} \right)_{i}+C_{V2}\sum V_{i}^{2}\left( I-I_{sc} \right)_{i}+C_{I1}\sum\left( I-I_{sc} \right)_{i}^{2}+C_{I2}\sum\left( I-I_{sc} \right)_{i}^{3}+C_{I1V1}\sum{V_{i}\left( I-I_{sc} \right)}_{i}^{2}=\sum\left( I-I_{sc} \right)_{i}{CC}_{i} (25)$$

$$C_{V0}\sum\left( I-I_{sc} \right)_{i}^{2} + C_{V1}\sum V_{i}{\left( I-I_{sc} \right)_{i}^{2}}_{i}+C_{V2}\sum V_{i}^{2}\left( I-I_{sc} \right)_{i}^{2}+C_{I1}\sum\left( I-I_{sc} \right)_{i}^{3}+C_{I2}\sum\left( I-I_{sc} \right)_{i}^{4}+C_{I1V1}\sum{V_{i}\left( I-I_{sc} \right)}_{i}^{3}=\sum\left( I-I_{sc} \right)_{i}^{2}{CC}_{i} (26)$$

$$C_{V0}\sum V_{i}\left( I-I_{sc} \right)_{i}+ C_{V1}\sum V_{i}^{2}\left( I-I_{sc} \right)_{i}+C_{V2}\sum V_{i}^{3}\left( I-I_{sc} \right)_{i}+C_{I1}\sum{V_{i}\left( I-I_{sc} \right)}_{i}^{2}+C_{I2}\sum{V_{i}\left( I-I_{sc} \right)}_{i}^{3}+C_{I1V1}\sum{V_{i}^{2}\left( I-I_{sc} \right)}_{i}^{2}=\sum{V_{i}\left( I-I_{sc} \right)}_{i}{CC}_{i} (27)$$

or in matrix notation

$\vec{V}=\hat{Mat}\vec{C}$ (28)

where the vector $\vec{C}$ and $\vec{V}$, and the matrix $\hat{Mat}$ have been defined as

$\vec{C}=\left( \begin{matrix} C_{V0} \\ C_{V1} \\ C_{V2} \\ C_{I1} \\ C_{I2} \\ C_{I1V1} \end{matrix} \right),$ (29)

$$\vec{V}=\left( \begin{matrix} \sum{CC}_{i} \\ \sum V_{i}{CC}_{i} \\ \sum V_{i}^{2}{CC}_{i} \\ \sum\left( I-I_{sc} \right)_{i}{CC}_{i} \\ \sum\left( I-I_{sc} \right)_{i}^{2}{CC}_{i} \\ \sum{V_{i}\left( I-I_{sc} \right)}_{i}{CC}_{i} \end{matrix} \right) (30)$$

$$\hat{Mat}=\left( \begin{matrix} N & \sum V_{i} & \sum V_{i}^{2} & \sum\left( I-I_{sc} \right)_{i} & \sum\left( I-I_{sc} \right)_{i}^{2} & \sum V_{i}\left( I-I_{sc} \right)_{i} \\ \sum V_{i} & \sum V_{i}^{2} & \sum V_{i}^{3} & \sum{V_{i}\left( I-I_{sc} \right)}_{i} & \sum V_{i}\left( I-I_{sc} \right)_{i}^{2} & \sum V_{i}^{2}\left( I-I_{sc} \right)_{i} \\ \sum V_{i}^{2} & \sum V_{i}^{3} & \sum V_{i}^{4} & \sum{V_{i}^{2}\left( I-I_{sc} \right)}_{i} & \sum V_{i}^{2}\left( I-I_{sc} \right)_{i}^{2} & \sum V_{i}^{3}\left( I-I_{sc} \right)_{i} \\ \sum\left( I-I_{sc} \right)_{i} & \sum{V_{i}\left( I-I_{sc} \right)}_{i} & \sum{V_{i}^{2}\left( I-I_{sc} \right)}_{i} & \sum\left( I-I_{sc} \right)_{i}^{2} & \sum\left( I-I_{sc} \right)_{i}^{3} & \sum{V_{i}\left( I-I_{sc} \right)}_{i}^{2} \\ \sum\left( I-I_{sc} \right)_{i}^{2} & \sum V_{i}\left( I-I_{sc} \right)_{i}^{2} & \sum V_{i}^{2}\left( I-I_{sc} \right)_{i}^{2} & \sum\left( I-I_{sc} \right)_{i}^{3} & \sum\left( I-I_{sc} \right)_{i}^{4} & \sum{V_{i}\left( I-I_{sc} \right)}_{i}^{3} \\ \sum V_{i}\left( I-I_{sc} \right)_{i} & \sum V_{i}^{2}\left( I-I_{sc} \right)_{i} & \sum V_{i}^{3}\left( I-I_{sc} \right)_{i} & \sum{V_{i}\left( I-I_{sc} \right)}_{i}^{2} & \sum{V_{i}\left( I-I_{sc} \right)}_{i}^{3} & \sum{V_{i}^{2}\left( I-I_{sc} \right)}_{i}^{2} \end{matrix} \right) (31)$$

All sums in Eqs 22 – 27, 30, and 31 are done from *i = 0, 1, …,*  $N-1$. The $\vec{C}$ vector is obtained solving Eq 28.

For clarity purposes, and as it is implemented in the program, the following standard statistical procedure to calculate $\Delta C_{V0}$, ${\Delta C}_{V1}$, ${\Delta C}_{V2}$, ${\Delta C}_{I1}$, $\Delta C_{I2}$, and $\Delta C_{V1I1}$ is exposed next. Further details can be found in [85, 86].

The matrix $\hat{W}$, and its transpose $\hat{W^{T}}$ are defined as

$$\hat{W}=\left( \begin{matrix} 1 & V_{0} & V_{0}^{2} & \left( I-I_{sc} \right)_{0} & \left( I-I_{sc} \right)_{0}^{2} & V_{0}\left( I-I_{sc} \right)_{0} \\ 1 & V_{1} & V_{1}^{2} & \left( I-I_{sc} \right)_{1} & \left( I-I_{sc} \right)_{1}^{2} & V_{1}\left( I-I_{sc} \right)_{1} \\ \vdots& \vdots& \vdots& \vdots& \vdots& \vdots\\ 1 & V_{N-1} & V_{N-1}^{2} & \left( I-I_{sc} \right)_{N-1} & \left( I-I_{sc} \right)_{N-1}^{2} & V_{N-1}\left( I-I_{sc} \right)_{N-1} \end{matrix} \right) , and (32)$$

$\hat{W^{T}}=\left( \begin{matrix} 1 & 1 & \ldots& 1 \\ V_{0} & V_{1} & \ldots& V_{N-1} \\ V_{0}^{2} & V_{1}^{2} & \ldots& V_{N-1}^{2} \\ \left( I-I_{sc} \right)_{0} & \left( I-I_{sc} \right)_{1} & \ldots& \left( I-I_{sc} \right)_{N-1} \\ \left( I-I_{sc} \right)_{0}^{2} & \left( I-I_{sc} \right)_{1}^{2} & \ldots& \left( I-I_{sc} \right)_{N-1}^{2} \\ V_{0}\left( I-I_{sc} \right)_{0} & V_{1}\left( I-I_{sc} \right)_{1} & \ldots& V_{N-1}\left( I-I_{sc} \right)_{N-1} \end{matrix} \right)$ (33)

Notice that $\hat{Mat}= \hat{W^{T}}\hat{W}$.

Next, the Hat matrix $\hat{Hat}$ is calculated as

$\hat{Hat}= {\hat{W}\left( \hat{W^{T}}\hat{W} \right)}^{-1}\hat{W^{T}}$ (34)

The trace of $\hat{Hat}$, i.e., $Tr\left( \hat{H}at \right)$, should, through this study, always have the value of 6, i.e., $Tr\left( \hat{Hat} \right)=6$, as one of the $\hat{H}at$ mathematical properties is to be the number of independent regression constants, which in this article is six, namely $C_{V0}, C_{V1}, C_{V2}, C_{I1}, C_{I2}, and C_{I1V1}$ [85, 86].

Utilizing the unitary matrix $\hat{1}$, the vector $\vec{E}$ is computed using

$\vec{E}=\left( \hat{1}-\hat{Hat} \right)\vec{CC}$ (35)

The parameter $\sigma$is computed using

$\sigma=\frac{\sum_{i=0}^{N-1} E_{i}^{2}}{N-Tr\left( \hat{Hat} \right)}$, (36)

where $E_{i}$ is each component of $\vec{E}$ (see Eq 35).

The covariance matrix $\hat{Cov}$ is computed using

$\hat{Cov}=\sigma\left( \hat{W^{T}}\hat{W} \right)^{-1}$. (37)

Eventually, $\Delta C_{V0}$, ${\Delta C}_{V1}$, ${\Delta C}_{V2}$, ${\Delta C}_{I1}$, $\Delta C_{I2}$, and $\Delta C_{V1I1}$ are calculated using

$\Delta C_{V0}=\sqrt{{Cov}_{0, 0}}$ (38)

$\Delta C_{V1}=\sqrt{{Cov}_{1,1}}$ (39)

$\Delta C_{V2}=\sqrt{{Cov}_{2,2}}$ (40)

$\Delta C_{I1}=\sqrt{{Cov}_{3,3}}$ (41)

$\Delta C_{I2}=\sqrt{{Cov}_{4,4}}$ (42)

$\Delta C_{V1I1}=\sqrt{{Cov}_{5,5}}$ (43)

where ${Cov}_{0, 0}$, ${Cov}_{1,1}, {Cov}_{2,2}, {Cov}_{3,3}, {Cov}_{4,4},$ and ${Cov}_{5,5}$ are the (0,0), (1,1), (2,2), (3,3), (4,4), and (5,5) components of the covariance matrix $\hat{Cov}$, respectively.

For the sake of clarity, the flow diagram of the program is shown in Fig S1, and a numerical computational example of the implementation of this procedure is given in Table S1, for the case of $N$= 9, using the file I-V9points.txt available in the Supplementary Material, and also shown in the Video, available also in the Supplementary Material. Before explaining the program CCSimpsonOrder5.exe, the conditions that the *IV* data file should have are commented next, to be correctly upload by the program. The *IV* data file should have the following characteristics (they are also in the Video with an example):


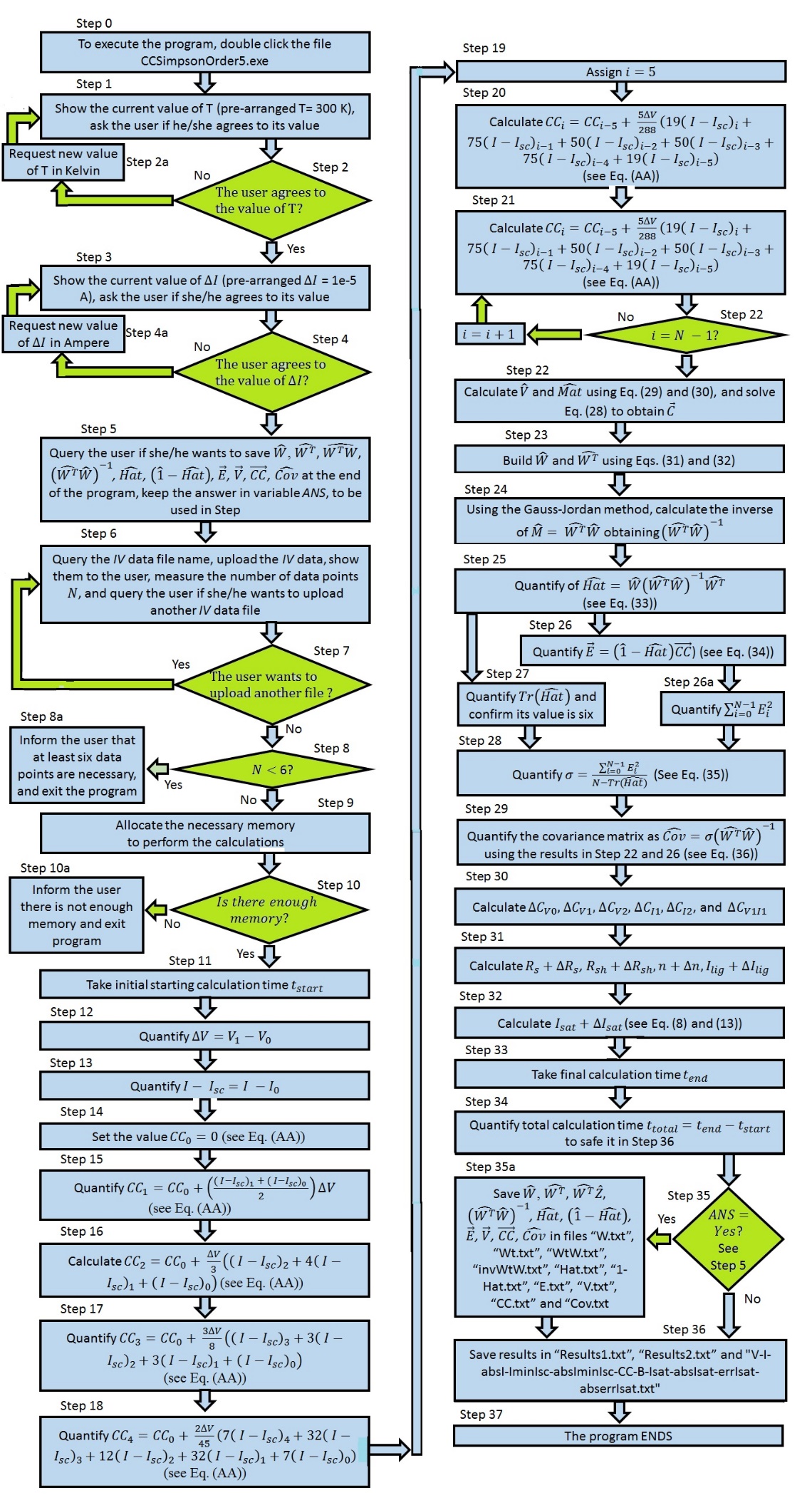


**S1** **Fig. Schematic diagram, on the program steps done to obtain** $\boldsymbol{R}_{\boldsymbol{s}}\boldsymbol{+\Delta}\boldsymbol{R}_{\boldsymbol{s}}$***,*** $\boldsymbol{R}_{\boldsymbol{sh}}\boldsymbol{+\Delta}\boldsymbol{R}_{\boldsymbol{sh}}\boldsymbol{, n+\Delta n,}\boldsymbol{I}_{\boldsymbol{lig}}\boldsymbol{+\Delta}\boldsymbol{I}_{\boldsymbol{lig}}\boldsymbol{,}$**and** $\boldsymbol{I}_{\boldsymbol{sat}}\boldsymbol{+\Delta}\boldsymbol{I}_{\boldsymbol{sat}}$**.**

1. The file name should have no spaces, and it should be a text file.
2. It should not have any headers. The first data line should be the voltage at 0 V and the current measured at 0 V. This current value will be taken by the program as $I_{sc}$.
3. Only two columns of data should be in the file, the first one is the voltage and the second one the current.
4. No additional characters should be before the first column of data starts, the first character should be part of the voltage number.
5. The voltage column and the current column should be separated by a Tab, whose Ascii code is 9, and in C++ is the \t character. The Tab let the program know when the voltage data column ends, and where the current data column starts.
6. There should not be any more characters at the end of the current data column, and an End Of Line (EOL) character should end the data line, allowing the program to know that the pair of *IV* data has ended. The Ascii code for EOL is 10, and in C++ is the \n character.
7. No more text should come at the end of *IV* data text file, and there should be an End Of File (EOF) character, informing the program that the *IV* data has ended. The Ascii code for EOF is 26.
8. $The voltage step, \Delta V$, should have the same value in the whole *IV* data file, as the program computes it as $\Delta V=$ $V_{1}- V_{0}$, and considers it constant.
9. Scientific notation is allowed, using the characters “e” or “E”. For example, “3.2e-3” or “4.1E2”, are the 0.0032 or 410 numbers, respectively. Expressions as “3.2×10^-3” or “4.1×10^2”, for the former examples, will not be recognizable by the program.
10. The program assumes that the voltage data are in Volts, and the current data are in Amperes, then, the extracted series and shunt resistances are given in Ohms, while the light and saturation current are given in Amperes.
11. The program assumes an increasing *IV* curve, i.e., if you graph it, it goes upward as voltage increases. In this case, the five deduced solar cell parameters will be extracted as positive numbers. In the case of a decreasing *IV* curves, this means, if you plot it, it goes downward as the voltage increases, all the five extracted solar cell parameters, except $n$, will be extracted as negative numbers, while $n$ will still be extracted as a positive number (see example in the Video in the Supplementary Material).

The program was written in C++. The double data type, which is an eight bytes long data type, was used in the program. Memory is dynamically allocated during the execution of the program, and it is liberated when the program ends. The user can estimate the necessary free memory bytes before executing the program, using the following equation:

$necessary memory=16N^{2}+112N+114$ in bytes (44)

The program was run in a Toshiba Laptop, model Satellite L55w-c, with processor Intel Core i7-5500U CPU, and 10 GB memory. The operative system was a 64 bits Windows 8. The author does not know if the program can be executed under other operative systems. The results reported in this article may be different in case the available memory, the processor, and/or the operative system are different.

To execute the program, double click the CCSimpsonOrder5.exe program. The program and your *IV* data text file should be in the same folder (Step 0 in Fig S1), where the program will save all the files. The program will be executed in a DOS prompt screen. A text commenting the article appears first, asking you to press any key. The program informs then that the *k* value is 8.6E-5 eV/K. An opportunity is then given to the user to change *T*, and $\Delta I$, which have 300 K and 1e-5 pre-arranged values (Step 1 to 4 in Fig S1). In Step 5 in Fig S1, the program asks the user if she/he wishes to save $\hat{W}$, $\hat{W^{T}}$, $\hat{W^{T}}\hat{W}$, $\left( \hat{W^{T}}\hat{W} \right)^{-1}$*,* $\hat{Hat}$*,* $\left( \hat{1}-\hat{Hat} \right)$*,* $\vec{E}$*,* $\hat{Cov}, \vec{CC}$*,* and $\vec{V}$in text files. The program performance time increases abruptly, in case the user agrees, as it takes time for the program to save this data. The *IV* data file name is asked in Step 6 in Fig S1, which should be in the same folder as the program. After reading the file, the program shows the *IV* text data to the user, and asks the user if she/he accepts to this *IV* file, in Step 7 in Fig S1. In case not, the program goes back to Step 6 and requests one more time the file name, repeating the former steps. In case the user accepts the *IV* data, the program confirms (Step 8 in Fig S1) if $N\geq6$, which is a necessary to do the calculations. In case $N<6$, it tells the user that her/his *IV* data file has less than 6 data points, that at least 6 data points are necessary to perform the computations, and the program exits (Step 8a in Fig S1). All these steps are explained in the Video available in the Supplementary Material.

Memory allocation is the next Step 9 in Fig S1. In case there is not enough memory (Step 10 in Fig S1) to execute the program, the program tells it to the user, and exits (Step 10a in Fig S1). An example is given in the Video in the Supplementary Material. Once the necessary memory is allocated, $\Delta V=$ $V_{1}- V_{0}$ and $I-I_{sc}={I-I}_{0}$ are calculated (Step 11 and 12 in Fig2).

A numerical example is given in Table S1 and in Fig S2, for the case of 9 data points, i.e., the file I-V9points.txt, which is also explained in the Video in the Supplementary Material. In Col 1 and Col 2 in Table I, the *IV* data are given. In Col 3, $I-I_{sc}$ is reported, computed as Col2 – (L1, Col2). The following steps, Step 13 to 19 in Fig S1, are the computation of $\vec{CC}$, using Eqs 14 – 19. This is shown in Col 4 in Table I and in Fig S2, where the implementation of Eqs 14 – 19 are given, and the final result of *CC(V, I)* is reported in Col. 5 in Table I. This example is also commented in the Video in the Supplementary Material. Then, for our numerical example of 9 data points (file I-V9points.txt)

**S1 Table**. Example on the application of program CCSimpsonOrder5.exe, on *IV* data file I-V9points.txt, available in the Supplementary Material. First, in Column 1 and Column 2, namely Col1 and Col2, the *IV* data from file I-V9points.txt, is given. Afterwards, in Col3, *I - I_sc_*, is reported, as the value of Column 2, minus the value (Line 1, Column 2), i.e., Col2 – (L1, Col2). Next, in Col4, an explanation on how *CC(V, I)* was calculated is given, using the trapezoidal integration method (Eq. (15)) in L2, the Newton Cotes integration (Eq. (16)) method in L3, the 3/8 rule (Eq. (17)) in L4, and then, from L5 to L8, the Boole’s integration formula (Eq. (18)), adding it to value in the previous line and same column is shown. For the sake of clarity, the numerical calculations are given. Finally, in Col5 the final value of *CC(V, I)* is reported.

| Line / Column | Col1 | Col2 | Col3 | Col4 | Col5 |
| --- | --- | --- | --- | --- | --- |
|  | Voltage  *V* (V) | Current  *I* (mA) | $f\left( V \right)=I-I_{sc}$(mA)  Col2 – (L1, Col2) | Explanation of which integration was used  to calculate *CC(V, I)* | *CC(V, I)*  *(mW)* |
| L1 | 0 | -0.999 | 0 | $\int_{0 V}^{0 V} f\left( V \right)=0 mW$ | 0 m*W* |
| L2 | 0.125 | -0.868 | 0.1309 | Trapezoidal integration is used in this case, adding it to the result in $\left( L1, Col5 \right)$, see Fig S2A  $\int_{0 V}^{0.125 V} f\left( V \right)\approx\left( L1, Col5 \right)+\Delta V\left( \frac{f\left( 0 V \right)+f\left( 0. 125 V \right)}{2} \right)=0 mW+\left( 0.125 V \right)\left( \frac{0 mA+0.1309 mA}{2} \right)=0.00818 mW$ | 0.00818 m*W* |
| L3 | 0.25 | -0.702 | 0.2974 | Newton Cotes integration is used in this case, adding it to the result in $\left( L1, Col5 \right)$, see Fig S2B  $\int_{0 V}^{0.25 V} f\left( V \right)\approx\left( L1, Col5 \right)+\left( \frac{\Delta V}{3} \right)\left( f\left( 0.25 V \right)+4f\left( 0. 125 V \right)+f\left( 0 V \right) \right)=0 mW+\left( \frac{0.125 V}{3} \right)\left( 0.2974 mA+4\left( 0.1309 mA \right)+0 mA \right)=0.03421 mW$ | $0.03421$ *mW* |
| L4 | 0.375 | -0.289 | 0.7097 | 3/8 integration is used in this case, adding it to the result in $\left( L1, Col5 \right)$, see Fig S2C  $\int_{0 V}^{0.375 V} f\left( V \right)\approx\left( L1, Col5 \right)+\left( \frac{3\Delta V}{8} \right)\left( f\left( 0.375 V \right)+3f\left( 0.25 V \right)++3f\left( 0.125 V \right)+f\left( 0 V \right) \right)= 0 mW+\left( \frac{3\times0.125 V}{8} \right)\left( 0.7097 mA+3\left( 0.2974 mA \right)+3\left( 0.1309 mA \right)+0 mA \right)=0.09349 mW$ | $0.09349$ *mW* |
| L5 | 0.5 | 1.761 | 2.7596 | Boole´s integration formula is used in this case, adding it to the result in $\left( L1, Col5 \right)$, see Fig S2D  $\int_{0 V}^{0.5 V} f\left( V \right)\approx\left( L1, Col5 \right)+\frac{2\Delta V}{45}\left( 7f\left( 0.5 V \right)+32f\left( 0.375 V \right)+12f\left( 0.25 V \right)+32f\left( 0.125 V \right)+7f\left( 0 V \right) \right)=0 mW+\frac{2\left( 0.125 V \right)}{45}\left( 7\times2.7596 mA+32\times0.7097 mA+12\times0.2974 mA+32\times0.1309 mA+7\times0 mA \right)=0.27658 mW$ | $0.27658 mW$ |
| L6 | 0.625 | 12.847 | 13.8466 | Order 5 Simpson integration formula is used in this case, adding it to the result in $\left( L1, Col5 \right)$, see Fig S2E  $\int_{0 V}^{0.625 V} f\left( V \right)\approx\left( L1, Col5 \right)+\frac{5\Delta V}{288}\left( 19f\left( 0.625 V \right)+75f\left( 0.5 V \right)+50f\left( 0.375 V \right)+50f\left( 0.25 V \right)+75f\left( 0.125 V \right)+19f\left( 0 V \right) \right)=0 mW+\frac{5\left( 0.125 V \right)}{288}\left( 19\times13.8466 mA+75\times2.7596 mA+50\times0.7097 mA+50\times0.2974 mA+75\times0.1309 mA+19\times0 mA \right)=1.15069 mW$ | $1.15069$*mW* |
| L7 | 0.75 | 50.763 | 51.7629 | Order 5 Simpson integration formula is used in this case, adding it to the result in $\left( L2, Col5 \right)$, see Fig S2F  $\int_{0 V}^{0.75 V} f\left( V \right)\approx\left( L2, Col5 \right)+\frac{5\Delta V}{288}\left( 19f\left( 0.75 V \right)+75f\left( 0.625 V \right)+50f\left( 0.5 V \right)+50f\left( 0.375 V \right)+75f\left( 0.25 V \right)+19f\left( 0.125 V \right) \right)=0.00818 mW+\frac{5\left( 0.125 V \right)}{288}\left( 19\times51.7629 mA+75\times13.8466 mA+50\times2.7596 mA+50\times0.7097 mA+75\times0.2974 mA+19\times0.1309 mA \right)=4.82645 mW$ | $4.82645 mW$ |
| L8 | 0.875 | 120.341 | 121.34 | Order 5 Simpson integration formula is used in this case, adding it to the result in $\left( L3, Col5 \right)$, see Fig S2G  $\int_{0 V}^{0.875 V} f\left( V \right)\approx\left( L3, Col5 \right)+\frac{5\Delta V}{288}\left( 19f\left( 0.875 V \right)+75f\left( 0.75 V \right)+50f\left( 0.625 V \right)+50f\left( 0.5 V \right)+75f\left( 0.375 V \right)+19f\left( 0.25 V \right) \right)=0.03421 mW+\frac{5\left( 0.125 V \right)}{288}\left( 19\times121.34 mA+75\times51.7629 mA+50\times13.8466 mA+50\times2.7596 mA+75\times0.7097 mA+19\times0.2974 mA \right)=15.392 mW$ | $15.392 mW$ |
| L9 | 1 | 209.615 | 210.614 | Order 5 Simpson integration formula is used in this case, adding it to the result in $\left( L4, Col5 \right)$, see Fig S2H  $\int_{0 V}^{1 V} f\left( V \right)\approx\left( L4, Col5 \right)+\frac{5\Delta V}{288}\left( 19f\left( 1 V \right)+75f\left( 0.875 V \right)+50f\left( 0.75 V \right)+50f\left( 0.625 V \right)+75f\left( 0.5 V \right)+19f\left( 0.375 V \right) \right)=0.09349 mW+\frac{5\left( 0.125 V \right)}{288}\left( 19\times210.614 mA+75\times121.34 mA+50\times51.7629 mA+50\times13.8466 mA+75\times2.7596 mA+19\times0.7097 mA \right)=36.1246 mW$ | $36.1246 mW$ |


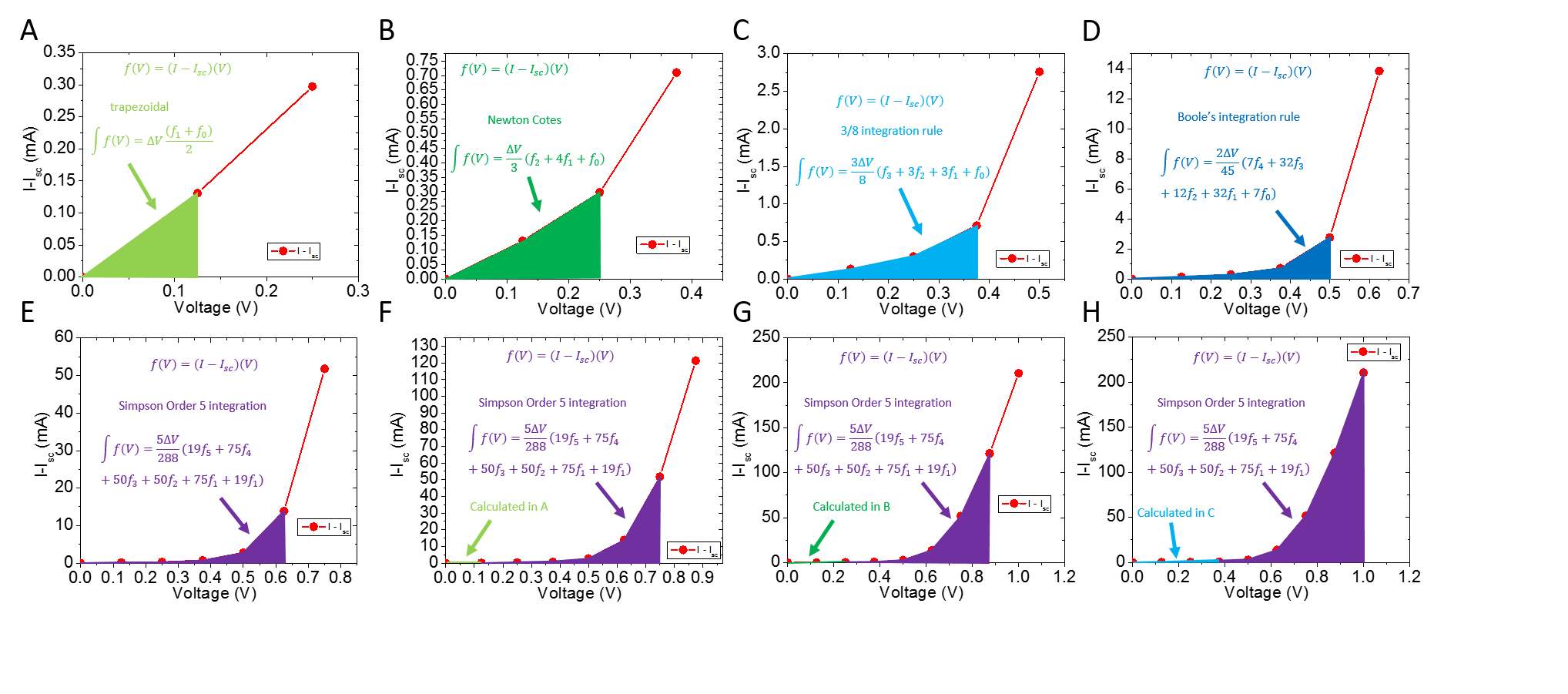


**S2 Fig.** Visual explanation, on how the $CC\left( V,I \right)$ is calculated in Table S1, in A using the trapezoidal integration, in B the Newton Cotes integration, in C, the 3/8 integration, in D the Boole’s integration, in E the order 5 Simpson integration, F the order 5 Simpson integration, in F the order 5 Simpson integration, adding it to the integration obtained in A, in G the order 5 Simpson integration, adding it to the integration obtained in B, and in H the order 5 Simpson integration, adding it to the integration obtained in C.

$\vec{CC}=\left( \begin{matrix} 0 \\ 8.18077e-006 \\ 3.42089e-005 \\ 9.35029e-005 \\ 0.000276592 \\ 0.00115069 \\ 0.00482645 \\ 0.015392 \\ 0.0361246 \end{matrix} \right)$ (45)

Once $\vec{CC}$ is computed, the $\vec{V}$ and $\hat{Mat}$ are computed using to Eqs 29 and 30 (Step 20 in Fig S1), obtaining

$\vec{V}=\left( \begin{matrix} 0.0579063 \\ 0.0541146 \\ 0.0511581 \\ 0.00974262 \\ 0.0018422 \\ 0.0094403 \end{matrix} \right),$ and (46)

$$\hat{Mat}=\left( \begin{matrix} 9 & 4.5 & 3.1875 & 0.401462 & 0.0619611 & 0.366 \\ 4.5 & 3.1875 & 2.53125 & 0.366 & 0.0593747 & 0.338851 \\ 3.1875 & 2.53125 & 2.1416 & 0.338851 & 0.0572149 & 0.317508 \\ 0.401462 & 0.366 & 0.338851 & 0.0619611 & 0.0112704 & 0.0593747 \\ 0.0619611 & 0.0593747 & 0.0572149 & 0.0112704 & 0.00219165 & 0.0110114 \\ 0.366 & 0.338851 & 0.317508 & 0.0593747 & 0.0110114 & 0.0572149 \end{matrix} \right) (47)$$

and $\vec{C}$ is calculated solving Eq 29 (Step 20 in Fig S1), obtaining

$\vec{C}=\left( \begin{matrix} 2.70367e-006 \\ -0.000137568 \\ 0.000738644 \\ 0.0539629 \\ 0.495648 \\ 0.0103051 \end{matrix} \right),$ (48)

The matrixes $\hat{W}$ and $\hat{W^{T}}$ are constructed next, using Eqs 32 and 33 (Step 21 in Fig S1), obtaining

$\hat{W}=\left( \begin{matrix} 1 & 0 & 0 & 0 & 0 & 0 \\ 1 & 0.125 & 0.015625 & 0.000130892 & 1.71328e-008 & 1.63615e-005 \\ 1 & 0.25 & 0.0625 & 0.000297444 & 8.84728e-008 & 7.43609e-005 \\ 1 & 0.5 & 0.25 & 0.00275966 & 7.61574e-006 & 0.00137983 \\ 1 & 0.625 & 0.390625 & 0.0138466 & 0.00019173 & 0.00865415 \\ 1 & 0.75 & 0.5625 & 0.0517629 & 0.0026794 & 0.0388222 \\ 1 & 0.875 & 0.765625 & 0.12134 & 0.0147235 & 0.106173 \\ 1 & 1 & 1 & 0.210614 & 0.0443582 & 0.210614 \end{matrix} \right)$, (49)

and

$\hat{W^{T}}=\left( \begin{matrix} 1 & 1 & 1 & 1 & 1 & 1 & 1 & 1 & 1 \\ 0 & 0.125 & 0.25 & 0.375 & 0.5 & 0.625 & 0.75 & 0.875 & 1 \\ 0 & 0.015625 & 0.0625 & 0.140625 & 0.25 & 0.390625 & 0.5625 & 0.765625 & 1 \\ 0 & 0.000130892 & 0.000297444 & 0.000709721 & 0.00275966 & 0.0138466 & 0.0517629 & 0.12134 & 0.210614 \\ 0 & 1.71328e-008 & 8.84728e-008 & 5.03704e-007 & 7.61574e-006 & 0.00019173 & 0.0026794 & 0.0147235 & 0.0443582 \\ 0 & 1.63615e-005 & 7.43609e-005 & 0.000266145 & 0.00137983 & 0.00865415 & 0.0388222 & 0.106173 & 0.210614 \end{matrix} \right)$ (50)

The matrix $\left( \hat{W^{T}}\hat{W} \right)^{-1}$ is computed next, using the Gauss-Jordan method to obtain the inverse of $\hat{W^{T}}\hat{W}$ (Step 22 in Fig S1), yielding

$\left( \hat{W^{T}}\hat{W} \right)^{-1}=\left( \begin{matrix} 0.898889 & -6.35418 & 10.5434 & -303.943 & 497.943 & 384.622 \\ -6.35418 & 80.8171 & -169.064 & 6418.24 & 10569.3 & -8194.44 \\ 10.5434 & -169.064 & 405.838 & -19766 & -33339.9 & 25610.3 \\ -303.943 & 6418.24 & -19766 & 1.50842e+006 & 2.68218e+006 & -2.00794e+006 \\ -497.943 & 10569.3 & -33339.9 & 2.68218e+006 & 4.81715e+006 & -3.58491e+006 \\ 384.622 & -8194.44 & 25610.3 & -2.00794e+006 & -3.58491e+006 & 2.67764e+006 \end{matrix} \right)$(51)

The computation of $\hat{Hat}= {\hat{W}\left( \hat{W^{T}}\hat{W} \right)}^{-1}\hat{W^{T}}$ (Eq 33) is next, in Step 23 in Fig S1, obtaining

$$\hat{Hat}=\left( \begin{matrix} 0.898889 & 0.235858 & -0.0925438 & -0.114866 & 0.0457837 & 0.0705425 & -0.0714267 & 0.0355649 & -0.00780173 \\ 0.235858 & 0.401016 & 0.359203 & 0.142722 & -0.113154 & -0.089284 & 0.106154 & -0.0546433 & 0.0121285 \\ -0.0925438 & 0.359203 & 0.481776 & 0.309433 & -0.0101857 & -0.0958942 & 0.0757916 & -0.0350488 & 0.00746901 \\ -0.114866 & 0.142722 & 0.309433 & 0.374277 & 0.298549 & 0.0482037 & -0.101083 & -0.101083 & -0.0124865 \\ 0.0457837 & -0.113154 & -0.0101857 & 0.298549 & 0.581267 & 0.31793 & -0.179808 & 0.0749354 & -0.0153173 \\ 0.0705425 & -0.089284 & -0.0958942 & 0.0482037 & 0.31793 & 0.543527 & 0.317984 & 0.143278 & 0.0302678 \\ -0.0714267 & 0.106154 & 0.0757916 & -0.101083 & -0.179808 & 0.317984 & 0.769577 & 0.105132 & -0.0223198 \\ 0.0355649 & -0.0546433 & -0.0350488 & 0.0552512 & 0.0749354 & -0.143278 & 0.105132 & 0.951848 & 0.0102382 \\ -0.00780173 & 0.0121285 & 0.00746901 & -0.0124865 & -0.0153173 & 0.0302678 & -0.0223198 & 0.0102382 & 0.997822 \end{matrix} \right)$$

$$(52)$$

Notice that the value of $Tr\left( \hat{H} \right)$ from Eq 52 is

$Tr\left( \hat{H} \right)=0.898889+0.401016+0.481776+0.374277+0.581267+0.543527+0.769577+0.951848 + 0.997822=6$ (53)

confirming that is 6 (Step 23a in Fig S1). The program shows the value of $Tr\left( \hat{H} \right) to the user$.

As Step 24 in Fig S1, the program calculates $\vec{E}=\left( \hat{1}-\hat{Hat} \right)\vec{CC}$ (see Eq 34), obtaining

$\vec{E}=\left( \begin{matrix} -2.70367e-006 \\ 3.89136e-006 \\ 2.87086e-006 \\ -2.7755e-006 \\ -8.90203e-006 \\ 1.40124e-005 \\ -9.94861e-006 \\ 4.51038e-006 \\ -9.55177e-007 \end{matrix} \right)$ (54)

computing then $\sum_{i=0}^{N_{points}-1} e_{i}^{2}=4.342216e-010$ (Step 25 in Fig S1). These results are used in Step 26 in Fig S1 using Eq 35, to obtain the value of $\sigma=1.447405e-010$ (see Eq 35), which, in this particular case exposed here, namely the case of 9 points, is the one third of $\sum_{i=0}^{N_{points}-1} e_{i}^{2}$, as $N-Tr\left( \hat{H} \right)=9-6=3$ (see Eq 35).

The covariance matrix computation is the next Step 27 in Fig S1, computed using Eq 36, yielding

$\hat{Cov}=\left( \begin{matrix} 1.30106e-010 & -9.19707e-010 & 1.52605e-009 & -4.39929e-008 & -7.20725e-008 & 5.56703e-008 \\ -9.19707e-010 & 1.16975e-008 & -2.44704e-008 & 9.2898e-007 & 1.52981e-006 & -1.18607e-006 \\ 1.52605e-009 & -2.44704e-008 & 5.87411e-008 & -2.86095e-006 & -4.82563e-006 & 3.70685e-006 \\ -4.39929e-008 & 9.2898e-007 & -2.86095e-006 & 0.00021833 & 0.00038822 & -0.00029063 \\ -7.20725e-008 & 1.52981e-006 & -4.82563e-006 & 0.00038822 & 0.000697237 & -0.000518882 \\ 5.56703e-008 & -1.18607e-006 & 3.70685e-006 & -0.00029063 & -0.000518882 & 0.000387563 \end{matrix} \right)$ (55)

The standard deviations $\Delta C_{V0}$, ${\Delta C}_{V1}$, ${\Delta C}_{V2}$, ${\Delta C}_{I1}$, $\Delta C_{I2}$, and $\Delta C_{V1I1}$, are then computed using Eqs 37 – 42 in Step 28 Fig S1.

The calculation of $R_{s}\pm\Delta R_{s}$*,* $R_{sh}\pm\Delta R_{sh}, n\pm\Delta n, I_{lig}\pm\Delta I_{lig}$ is the next Step 29 in Fig S1, using Eqs 4 – 7, 9 – 12, and the results obtained in Steps 20 and 28 in Fig S1

The results obtained in Step 9 in Fig S1 are used to calculate $B=\left( exp((V-IR_{s})/nkT)-1 \right)^{-1}$ , $I_{sat}\pm\Delta I_{sat}$ (see Eqs 8 and 13), as $\left| I_{sat} \right|$ and $\left| \Delta I_{sat} \right|,$as Step 30 in Fig S1.

In Step 31 in Fig S1, the program confirms if the answer was “Yes” in Step 5, then $\hat{W}$, $\hat{W^{T}}$, $\hat{W^{T}}\hat{W}$, $\left( \hat{W^{T}}\hat{XW} \right)^{-1}$*,* $\hat{Hat}$*,* $\left( \hat{1}-\hat{Hat} \right)$*,* $\vec{E}$*,* $\vec{CC}$, and $\vec{V}$ are saved in the files “W.txt”, “Wt.txt”, “WtW.txt”, “inv(WtW).txt”, “Hat.txt”, “1-Hat.txt”, “E.txt”, “CC.txt”, “Cov.txt”, and “V.txt”, respectively.

The program saves the results in “Results1.txt”, “Results2.txt” and "V-I-absI-IminIsc-absIminIsc-CC-B-Isat-absIsat-errIsat-abserrIsat.txt".

In “Results1.txt”, the $C_{V0}$, ${\Delta C_{V0}, C}_{V1}$, ${\Delta C}_{V1}$, $C_{V2}$, ${{\Delta C}_{V2}, C}_{I1}$, ${\Delta C}_{I1}$, $C_{I2}$, $\Delta C_{I2}$*,* $C_{V1I1}, \Delta C_{V1I1}$, $R_{s}, \Delta R_{s}$*,* $R_{sh}, \Delta R_{sh},n, \Delta n,I_{lig}, \Delta I_{lig}, I_{sat}, \Delta I_{sat}$ are written tab.separated in different columns, to allow the user to easily import them into other softwares, such as Excel or Origin, while in “Results2.txt”, they are written separated by tabs in columns as $C_{V0}\pm{\Delta C_{V0}, C}_{V1}\pm{\Delta C}_{V1}$, $C_{V2}\pm{{\Delta C}_{V2}, C}_{I1}\pm{\Delta C}_{I1}$, $C_{I2}\pm\Delta C_{I2}$*,* $C_{V1I1} \pm\Delta C_{V1I1}$, $R_{s}\pm\Delta R_{s}$*,* $R_{sh}\pm\Delta R_{sh}, n\pm\Delta n, I_{lig}\pm\Delta I_{lig}, I_{sat}\pm\Delta I_{sat}$, to allow the user to easily copy-paste them when writing his/her manuscript in software like Word. Examples are given in the Video in the Supplementary Material.

Finally, in "V-I-absI-IminIsc-absIminIsc-CC-B-Isat-absIsat-errIsat-abserrIsat.txt", the *V*, *I*, $\left| I \right|$, $I-I_{sc}$, $\left| I-I_{sc} \right|$, *CC*, *B*, $I_{sat}, \left| I_{sat} \right|, \Delta I_{sat}$, and $\left| {\Delta I}_{sat} \right|$ are written in a text file, also in columns that are tab-separated, to make it easy for the user to import them to tables, like in Excell or Origin. In all these written results files, the total calculation time and $N$ are reported.

The program was run in *IV* data files containing 4, 6, 7, 8, 9, 10, 11, 19, 26, 38, 51, 64, 76, 101, 251, 501, 751, 1001, 2501, 5001, 7501, 10001, 12501, 15001, 17501, and 20001 data points, namely the files IV-4points.txt, IV-6points.txt, IV-7points.txt, IV-8points.txt, IV-9points.txt, IV-10points.txt, IV-11points.txt, IV-19points.txt, IV-26points.txt, IV-38points.txt, IV-51points.txt, IV-64points.txt, IV-76points.txt, IV-101points.txt, IV-251points.txt, IV-501points.txt, IV-751points.txt, IV-1001points.txt, IV-2501points.txt, IV-5001points.txt, IV7-501points.txt, IV-10001points.txt, IV-12501points.txt, IV-15001points.txt, IV-17501points.txt, and IV-20001points.txt, respectively, and also with the files where a $p_{n}=0.01 \%$, 0.05 %, and 0.1 % of noise was added provided in the Supplementary Material, and as explained before. Comparison was done with the results obtained using the trapezoidal integration method, to evaluate the improvement on the extraction of the five solar cell parameters.

The results obtained for former *IV* files are reported and commented in the next Section.

A summary of the application of the program is given in Table S2, showing the $C_{V0} \pm{\Delta C}_{V0},$ $C_{V1}\pm{\Delta C}_{V1}, {C_{V2}\pm{\Delta C}_{V2},C}_{I1}\pm{\Delta C}_{I1}, C_{I2}\pm\Delta C_{I2}$, and $C_{V1I1}\pm{\Delta C}_{V1I1},$ together with the extracted $R_{s}\pm\Delta R_{s}$*,* $R_{sh}\pm\Delta R_{sh}, n\pm\Delta n, I_{lig}\pm\Delta I_{lig}, I_{sat}\pm\Delta I_{sat}$, in the case of noiseless *IV* curves.

**Table S2**. $C_{V0} \pm{\Delta C}_{V0},$ $C_{V1}\pm{\Delta C}_{V1}, {C_{V2}\pm{\Delta C}_{V2},C}_{I1}\pm{\Delta C}_{I1}, C_{I2}\pm\Delta C_{I2}$*,* $C_{V1I1}\pm{\Delta C}_{V1I1}, R_{s}\pm\Delta R_{s}$*,* $R_{sh}\pm\Delta R_{sh}, n\pm\Delta n, I_{lig}\pm\Delta I_{lig}, \mathrm{and} I_{sat}\pm\Delta I_{sat}$ obtained using the program CCSimpsonOrder5.exe. The value “nan” means “not-a-number”, and it appears in
$N=6$ case, as the standard deviation is undetermined, as explained in the text (see Eq. (35)).

| $C_{V0} \pm{\Delta C}_{V0},$ $C_{V1}\pm{\Delta C}_{V1}, {C_{V2}\pm{\Delta C}_{V2},C}_{I1}\pm{\Delta C}_{I1}, C_{I2}\pm\Delta C_{I2}$*,* and $C_{V1I1}\pm{\Delta C}_{V1I1}$ | | | | | | |
| --- | --- | --- | --- | --- | --- | --- |
| $N$ | $C_{V0} \pm{\Delta C}_{V0}$ | $C_{V1}\pm\Delta C_{V1}$ | $C_{V2}\pm\Delta C_{V2}$ | $C_{I1}\pm\Delta C_{I1}$ | $C_{I2}\pm\Delta C_{I2}$ | $C_{V1I1}\pm{\Delta C}_{V1I1}$ |
| 6 | -2.33299e-016 ± nan | -0.000206906 ± nan | 0.000424311 ± nan | 0.271645 ± nan | 0.982413 ± nan | -0.307694 ± nan |
| 7 | 2.34748e-007 ± 1.27354e-006 | -0.000134572 ± 1.28898e-005 | 0.000582316 ± 3.12309e-005 | 0.129506 ± 0.00216099 | 0.675083 ± 0.003902 | -0.101549 ± 0.00289438 |
| 8 | 1.29233e-006 ± 4.14978e-006 | -0.000114062 ± 4.00318e-005 | 0.000688908 ± 9.13193e-005 | 0.0575262 ± 0.00572977 | 0.504299 ± 0.0102677 | 0.00519762 ± 0.00764541 |
| 9 | 4.62392e-006 ± 1.66578e-005 | -0.000169591 ± 0.000157949 | 0.000920198 ± 0.00035395 | 0.0178221 ± 0.0215788 | 0.416255 ± 0.0385621 | 0.0621367 ± 0.0287502 |
| 10 | 3.54664e-006 ± 1.12189e-005 | -0.000136319 ± 0.000105056 | 0.000785064 ± 0.000232217 | 0.0304424 ± 0.0138314 | 0.43611 ± 0.0246791 | 0.045667 ± 0.018409 |
| 11 | 1.81604e-006 ± 3.69025e-006 | -9.86538e-005 ± 3.42037e-005 | 0.000640022 ± 7.48032e-005 | 0.04611 ± 0.00438645 | 0.464682 ± 0.00782479 | 0.0244616 ± 0.00583539 |
| 19 | -9.89992e-008 ± 2.22197e-007 | -6.39231e-005 ± 1.98219e-006 | 0.00049526 ± 4.18091e-006 | 0.064957 ± 0.000235089 | 0.501239 ± 0.000421515 | -0.00152944 ± 0.000312765 |
| 26 | -3.53897e-009 ± 1.51521e-008 | -6.54472e-005 ± 1.33399e-007 | 0.000499843 ± 2.782e-007 | 0.0645679 ± 1.55021e-005 | 0.5005 ± 2.79074e-005 | -0.0010019 ± 2.06386e-005 |
| 38 | -8.78605e-010 ± 1.56975e-009 | -6.55012e-005 ± 1.36682e-008 | 0.000499958 ± 2.82484e-008 | 0.064569 ± 1.56539e-006 | 0.500507 ± 2.83016e-006 | -0.00100478 ± 2.08583e-006 |
| 51 | -4.17962e-010 ± 1.94184e-009 | -6.55095e-005 ± 1.68068e-008 | 0.000499981 ± 3.45702e-008 | 0.0645676 ± 1.91142e-006 | 0.500504 ± 3.46509e-006 | -0.00100289 ± 2.54834e-006 |
| 64 | -3.58354e-010 ± 1.51026e-009 | -6.55107e-005 ± 1.30251e-008 | 0.000499985 ± 2.67183e-008 | 0.0645671 ± 1.47571e-006 | 0.500503 ± 2.67979e-006 | -0.00100215 ± 1.96816e-006 |
| 76 | -3.50832e-010 ± 6.96514e-010 | -6.55112e-005 ± 5.99387e-009 | 0.000499985 ± 1.22747e-008 | 0.0645675 ± 6.77581e-007 | 0.500504 ± 1.23179e-006 | -0.00100283 ± 9.03906e-007 |
| 101 | -5.35137e-010 ± 1.19855e-009 | -6.55089e-005 ± 1.02845e-008 | 0.000499979 ± 2.10156e-008 | 0.0645678 ± 1.15937e-006 | 0.500505 ± 2.11081e-006 | -0.00100318 ± 1.54713e-006 |
| 251 | -2.0776e-010 ± 7.08676e-010 | -6.55132e-005 ± 6.04938e-009 | 0.000499992 ± 1.2314e-008 | 0.0645665 ± 6.78789e-007 | 0.500502 ± 1.23939e-006 | -0.00100138 ± 9.06386e-007 |
| 501 | -9.29654e-011 ± 4.76848e-010 | -6.55145e-005 ± 4.06337e-009 | 0.000499996 ± 8.26085e-009 | 0.0645659 ± 4.55288e-007 | 0.500501 ± 8.32139e-007 | -0.00100055 ± 6.08081e-007 |
| 751 | 5.09865e-012 ± 4.00148e-010 | -6.55157e-005 ± 3.4078e-009 | 0.0005 ± 6.92517e-009 | 0.0645656 ± 3.81656e-007 | 0.5005 ± 6.97798e-007 | -0.00100008 ± 5.09777e-007 |
| 1001 | -1.53769e-010 ± 3.18157e-010 | -6.55138e-005 ± 2.70874e-009 | 0.000499994 ± 5.50342e-009 | 0.0645662 ± 3.03295e-007 | 0.500501 ± 5.54623e-007 | -0.00100096 ± 4.05127e-007 |
| 2501 | -7.20198e-011 ± 2.09737e-010 | -6.55148e-005 ± 1.78474e-009 | 0.000499997 ± 3.62473e-009 | 0.0645658 ± 1.99753e-007 | 0.500501 ± 3.65393e-007 | -0.00100046 ± 2.66839e-007 |
| 5001 | -3.63061e-011 ± 1.49476e-010 | -6.55152e-005 ± 1.27173e-009 | 0.000499999 ± 2.58251e-009 | 0.0645657 ± 1.42317e-007 | 0.5005 ± 2.60356e-007 | -0.00100022 ± 1.90117e-007 |
| 7501 | 2.12024e-011 ± 1.20038e-010 | -6.55159e-005 ± 1.02121e-009 | 0.000500001 ± 2.07369e-009 | 0.0645654 ± 1.14276e-007 | 0.5005 ± 2.09066e-007 | -0.000999902 ± 1.5266e-007 |
| 10001 | 1.7193e-011 ± 1.06116e-010 | -6.55158e-005 ± 9.02751e-010 | 0.000500001 ± 1.83311e-009 | 0.0645654 ± 1.01018e-007 | 0.5005 ± 1.84813e-007 | -0.000999889 ± 1.34949e-007 |
| 12501 | 2.97307e-011 ± 9.44102e-011 | -6.5516e-005 ± 8.03149e-010 | 0.000500001 ± 1.63084e-009 | 0.0645654 ± 8.98714e-008 | 0.5005 ± 1.64422e-007 | -0.000999821 ± 1.20058e-007 |
| 15001 | 2.48106e-011 ± 8.58084e-011 | -6.55159e-005 ± 7.29965e-010 | 0.000500001 ± 1.48222e-009 | 0.0645654 ± 8.16814e-008 | 0.5005 ± 1.49439e-007 | -0.000999825 ± 1.09118e-007 |
| 17501 | -4.17708e-011 ± 8.05971e-011 | -6.55151e-005 ± 6.85627e-010 | 0.000499998 ± 1.39218e-009 | 0.0645657 ± 7.67197e-008 | 0.5005 ± 1.40362e-007 | -0.00100024 ± 1.02489e-007 |
| 20001 | 2.36059e-011 ± 7.53082e-011 | -6.55159e-005 ± 6.40632e-010 | 0.000500001 ± 1.30081e-009 | 0.0645654 ± 7.16844e-008 | 0.5005 ± 1.31151e-007 | -0.000999877 ± 9.5763e-008 |
| Solar cell parameters obtained using the program, and the previous values of $C_{V0} \pm{\Delta C}_{V0},$ $C_{V1}\pm{\Delta C}_{V1}, {C_{V2}\pm{\Delta C}_{V2},C}_{I1}\pm{\Delta C}_{I1}, C_{I2}\pm\Delta C_{I2}$*, and* $C_{V1I1}\pm{\Delta C}_{V1I1}$ | | | | | | |
| $N$ | $R_{s}\pm\Delta R_{s}$ $(Ω$) | $R_{sh}\pm\Delta R_{sh} (Ω$) | $n\pm\Delta n$ | $I_{lig}\pm\Delta I_{lig} (A)$ | $I_{sat}\pm\Delta I_{sat} (A)$ | |
| 6 | 1.96156 ± nan | 1178.38 ± nan | 10.5131 ± nan | 0.000977375 ± nan | 0.0268011 ± nan | |
| 7 | 1.34805 ± 0.0153329 | 858.64 ± 46.0508 | 5.01257 ± 0.0843922 | 0.00098451 ± 2.34105e-005 | 0.000812972 ± 4.17692e-005 | |
| 8 | 1.0072 ± 0.0405875 | 725.786 ± 96.2076 | 2.22525 ± 0.223556 | 0.00103533 ± 5.82526e-005 | 2.23597e-007 ± 1.08041e-007 | |
| 9 | 0.831238 ± 0.152803 | 543.362 ± 209.001 | 0.685313 ± 0.84097 | 0.00113756 ± 0.000209668 | 1.079e-021 ± 1.49715e-020 | |
| 10 | 0.871028 ± 0.0977444 | 636.89 ± 188.388 | 1.17534 ± 0.539386 | 0.00108906 ± 0.000140509 | 4.06841e-013 ± 1.36871e-012 | |
| 11 | 0.92826 ± 0.0309677 | 781.223 ± 91.3063 | 1.78366 ± 0.171188 | 0.00103992 ± 4.65833e-005 | 5.20003e-009 ± 2.70202e-009 | |
| 19 | 1.00148 ± 0.00166598 | 1009.57 ± 8.52263 | 2.51523 ± 0.00918683 | 0.000999622 ± 2.75039e-006 | 1.07193e-006 ± 1.69766e-008 | |
| 26 | 0.999999 ± 0.000110296 | 1000.32 ± 0.556751 | 2.50009 ± 0.000605886 | 0.00100095 ± 1.84304e-007 | 9.90915e-007 ± 1.14664e-009 | |
| 38 | 1.00001 ± 1.11853e-005 | 1000.08 ± 0.0565062 | 2.50014 ± 6.11893e-005 | 0.00100099 ± 1.8829e-008 | 9.91171e-007 ± 2.12492e-010 | |
| 51 | 1.00001 ± 1.36947e-005 | 1000.04 ± 0.0691459 | 2.50008 ± 7.472e-005 | 0.00100099 ± 2.31183e-008 | 9.90883e-007 ± 2.35751e-010 | |
| 64 | 1.00001 ± 1.05911e-005 | 1000.03 ± 0.0534398 | 2.50006 ± 5.76894e-005 | 0.001001 ± 1.79015e-008 | 9.90781e-007 ± 2.06512e-010 | |
| 76 | 1.00001 ± 4.86833e-006 | 1000.03 ± 0.0245509 | 2.50008 ± 2.64889e-005 | 0.001001 ± 8.23379e-009 | 9.90863e-007 ± 1.52914e-010 | |
| 101 | 1.00001 ± 8.3425e-006 | 1000.04 ± 0.0420348 | 2.50009 ± 4.53248e-005 | 0.00100099 ± 1.41188e-008 | 9.90925e-007 ± 1.85335e-010 | |
| 251 | 1 ± 4.8985e-006 | 1000.02 ± 0.0246288 | 2.50004 ± 2.65378e-005 | 0.001001 ± 8.29553e-009 | 9.90661e-007 ± 1.53017e-010 | |
| 501 | 1 ± 3.28893e-006 | 1000.01 ± 0.0165219 | 2.50002 ± 1.78001e-005 | 0.001001 ± 5.57012e-009 | 9.90542e-007 ± 1.37975e-010 | |
| 751 | 1 ± 2.75797e-006 | 1000 ± 0.0138503 | 2.5 ± 1.49214e-005 | 0.001001 ± 4.67091e-009 | 9.90469e-007 ± 1.33014e-010 | |
| 1001 | 1 ± 2.19209e-006 | 1000.01 ± 0.0110071 | 2.50003 ± 1.18578e-005 | 0.001001 ± 3.71253e-009 | 9.906e-007 ± 1.27758e-010 | |
| 2501 | 1 ± 1.44419e-006 | 1000.01 ± 0.00724954 | 2.50001 ± 7.8097e-006 | 0.001001 ± 2.44586e-009 | 9.90529e-007 ± 1.20783e-010 | |
| 5001 | 1 ± 1.02904e-006 | 1000 ± 0.00516506 | 2.50001 ± 5.56413e-006 | 0.001001 ± 1.74276e-009 | 9.90494e-007 ± 1.16914e-010 | |
| 7501 | 0.999999 ± 8.26317e-007 | 999.999 ± 0.00414738 | 2.5 ± 4.46784e-006 | 0.001001 ± 1.39943e-009 | 9.90444e-007 ± 1.15022e-010 | |
| 10001 | 1 ± 7.3046e-007 | 999.999 ± 0.0036662 | 2.5 ± 3.94948e-006 | 0.001001 ± 1.23709e-009 | 9.90447e-007 ± 1.1413e-010 | |
| 12501 | 0.999999 ± 6.49866e-007 | 999.998 ± 0.00326166 | 2.5 ± 3.51368e-006 | 0.001001 ± 1.1006e-009 | 9.90437e-007 ± 1.13379e-010 | |
| 15001 | 1 ± 5.90648e-007 | 999.998 ± 0.00296443 | 2.5 ± 3.19348e-006 | 0.001001 ± 1.00031e-009 | 9.90438e-007 ± 1.12828e-010 | |
| 17501 | 1 ± 5.54772e-007 | 1000 ± 0.00278438 | 2.50001 ± 2.99949e-006 | 0.001001 ± 9.39547e-010 | 9.90498e-007 ± 1.125e-010 | |
| 20001 | 1 ± 5.18363e-007 | 999.998 ± 0.00260162 | 2.5 ± 2.80263e-006 | 0.001001 ± 8.77887e-010 | 9.90445e-007 ± 1.12156e-010 | |

^85^M. Abramowitz, and I. A. Stegun, *Handbook of Mathematical Functions with Formulas, Graphs, and Mathematical Tables*, 9^th^. ed. (Dover, New York, 1972).

^86^A. Basilevsky, *Applied Matrix Algebra in Statistical Sciences*, 1^st^. ed. (Dover, New York, 2005).

^87^D. A. Freedman, *Statistical Models: Theory and Practice*, 2^nd^. ed. (Cambridge, UK, 2009).
