# Supplementary material for: Five solar cell parameters automatic extraction, within the one diode-solar cell model, using the implemented Simpson order 5 integration method, in an executable program
Source: PLoS One. 2026 Apr 22;21(4):e0346051. doi: 10.1371/journal.pone.0346051 (PMC13102238; doi:10.1371/journal.pone.0346051)
Supplement: S2 File — Pseudo-code of program CCSimpsonOrder5.exe. (DOCX) [file pone.0346051.s002.docx]

**Pseudo-code of program CCSimpsonOrder5.exe**

STARTPROGRAM

DECLARE

xdata[]; ydata[]; helpxdata[]; helpydata[]; auxX[]; auxtranspX[]; auxA[][]; Hat[][]; IminHat[][]; auxE[]; IminIsc[]; CC[]; absI[]; absIminIsc[]; B[]; Isat[]; absIsat[]; errIsat[]; abserrIsat[]; m[6][6]; maux[6][6]; mident[6][6]; mult[6][6]; maux2[6][6]; covariance[6][6]; v[6];

num; i; i1; i2; i3; i4; i5; i6; Isc; deltax; det; deta; detb; detc; detd; dete; detf; det2; det3; det4; det5; det6; a; b; c; d; e; f; factor1; factor2; traceH; Sumae2; Sig2; vara; varb; varc; vard; vare; varf; dstda; dstdb; dstdc; dstdd; dstde; dstdf; Rs; Rsh; Ilig; n; A; IsatMaxV; errIsatMaxV; errRs; errRsh; errIlig; errn; sumx; sumx2; sumx3; sumx4; sumy; sumy2; sumy3; sumy4; sumxy; sumxy2; sumx2y; sumx3y; sumxy3; sumx2y2; sumz; sumxz; sumx2z; sumyz; sumy2z; sumxyz; Cv0; Cv1; Cv2; Ci1; Ci2; Cv1i1; errCv0; errCv1; errCv2; errCi1; errCi2; errCv1i1; k; T; Vth; errI; aux1; aux2; car; safematrixes; Vth = k*T;

ALLOCATE sumx=sumx2=sumx3=sumx4=sumy=sumy2=sumy3=sumy4=sumxy=sumxy2=sumx2y=sumx3y=sumxy3=sumx2y2=0;

ALLOCATE sumz=sumxz=sumx2z=sumyz=sumy2z=sumxyz=0;

ALLOCATE k=8.6e-5;

ALLOCATE errI = 1e-5

ALLOCATE num = 0;

ALLOCATE T = 300;

ALLOCATE Vth = k*T;

DISPLAY "You are running the program CCSimpsonOrder5.exe”;

DISPLAY “Further details about the program can be found from the article: ";

DISPLAY " Five solar cell parameters automatic extraction, within the one diode-solar cell model, using the implemented Simpson Order 5 integration method, in an executable program. Victor-Tapio Rangel-Kuoppa";

DISPLAY " Submitted to PLoS One (2025)";

DISPLAY "Press any key";

GETCHARACTER;

DISPLAY "The Boltzman constant value is k = %E eV/K", k;

DISPLAY " The pre-assigned absolute temperature T is T = %E K, do you want to change it? ", T;

DISPLAY "Press Y or y to change it";

DISPLAY “Press any other key to not change it and continue with the program";

ALLOCATE car = 'n';

car = GETCHARACTER;

DOWHILE car = 'y' OR car='Y'

DISPLAY "Give the new absolute temperature T in Kelvins T = ";

READ T;

DISPLAY "The new absolute temperature is T = %E K, doyou want to change it?", T;

DISPLAY "Press Y or y to change it”;

DISPLAY "Press any other key to not change it and continue with the program";

car = GETCHARACTER;

ENDWHILE

DISPLAY " The pre-assigned precision in I is deltaI = %E A, do you want to change it? ", errI;

DISPLAY "Press Y or y to change it";

DISPLAY "Press any other key to not change it and continue with the program";

ALLOCATE car = 'n';

car = GETCHARACTER;

DOWHILE car = 'y' OR car='Y'

DISPLAY "Give the new precision in I in Amperes deltaI = ";

READ errI;

DISPLAY "The new precision in I is deltaI = %E A, do you want to change it?", errI;

DISPLAY "Press Y or y to change it";

DISPLAY "Press any other key to not change it and continue with the program";

car = GETCHARACTER;

ENDWHILE

DISPLAY "Do you want the W, Wt, Mat = WtW, inv(WtW), Hat, 1-Hat, E, V, CoC, and Covariance matrixes to be saved after all the calculations?";

DISPLAY "Press Y or y to WRITEINFILE them";

DISPLAY "Press N or n to NOT WRITEINFILE them";

ALLOCATE safematrixes = 'a';

ALLOCATE safematrixes = GETCHARACTER;

DOWHILE safematrixes DIFFERENT y AND safematrixes DIFFERENT Y AND safematrixes DIFFERENT n AND safematrixes DIFFERENT N

DISPLAY "Do you want the W, Wt, Mat = WtW, inv(WtW), Hat, 1-Hat, E, V, CoC, and Covariance matrixes to be saved after all the calculations?";

DISPLAY "Press Y or y to WRITEINFILE them";

DISPLAY "Press N or n to NOT WRITEINFILE them";

safematrixes = GETCHARACTER;

ENDWHILE

DO

ALLOCATE car = 'a';

ALLOCATE num = 0; flag=0;

ALLOCATE xdata = ydata = helpxdata = helpydata = NULL;

DISPLAY "Enter file name (Do NOT forget to write the extension file, too!): ";

READ filename;

DISPLAY "You entered: ", filename;

OPEN filename;

READFILE filename SEPARATEIN xdata AND ydata;

ALLOCATE num = NUMBEROFDATAIN xdata;

CLOSE filename;

DISPLAY "Next the V vs I data are shown”

DISPLAY “Press any key ";

GETCHARACTER;

DISPLAY "V I";

STARTFOR i1=0 DOWHILE i1<num AND ALLOCATE i1 = i1+1

DISPLAY "%E %E", xdata[i1], ydata[i1];

ENDFOR

DISPLAY "Do you agree with this file or do you want to upload another file?";

DISPLAY "Press 'N' or 'n' to not agree and to read another file";

DISPLAY "Press any other key to accept the file and continue the program";

car = GETCHARACTER;

DOWHILE car='N' OR car='n';

STARTIF num < 6

THEN

DISPLAY "Your number of data points is N = %d, smaller than six", nu;

DISPLAY "It is necessary to have at least six different data points to run the program";

DISPLAY "The program exits now”;

DISPLAY “Press any key ";

GETCHARACTER;

return 0;

ENDIF;

DISPLAY "The computer will take the current time in seconds, after you press any key, to calculate the Total Calculation Time”

DISPLAY “Press any key ";

GETCHARACTER;

GETTIME time1;

DISPLAY "The total number of pair of data is N = %d", num;

ALLOCATEMEMORY to auxX WITH 6*num BYTES;

STARTIF auxX = NULL

THEN

DISPLAY "Not enough memory, the program exits now, press any key"; GETCHARACTER;

ENDIF;

ALLOCATEMEMORY to auxtranspX WITH 6*num BYTES;

STARTIF auxtranspX = NULL

THEN

DISPLAY "Not enough memory, the program exits now, press any key";

GETCHARACTER;

ENDIF;

ALLOCATEMEMORY to auxA WITH 6*num BYTES;

STARTIF auxA = NULL

THEN

DISPLAY "Not enough memory, the program exits now, press any key";

GETCHARACTER;

ENDIF;

ALLOCATEMEMORY to Hat WITH num*num BYTES;

STARTIF Hat = NULL

THEN

DISPLAY "Not enough memory, the program exits now, press any key";

GETCHARACTER;

ENDIF;

ALLOCATEMEMORY to IminHat WITH num*num BYTES;

STARTIF IminHat = NULL

THEN

DISPLAY "Not enough memory, the program exits now, press any key";

GETCHARACTER;

ENDIF;

ALLOCATEMEMORY to auxE WITH num BYTES;

STARTIF auxE = NULL

THEN

DISPLAY "Not enough memory, the program exits now, press any key";

GETCHARACTER;

ENDIF;

ALLOCATE Isc = ydata[0];

DISPLAY "The short circuit current value is Isc = %E A", Isc;

ALLOCATE deltax = xdata[1]-xdata[0];

DISPLAY "The delta voltage is deltaV = %E V\n, deltax;

DISPLAY "Now, I-Isc is built";

ALLOCATEMEMORY to IminIsc WITH num BYTES;

STARTIF IminIsc = NULL

THEN

DISPLAY "Not enough memory, the program exits now, press any key";

GETCHARACTER;

ENDIF;

STARTFOR i=0 DOWHILE i<num AND ALLOCATE i1 = i1+1

IminIsc[i]=ydata[i]-Isc;

ENDFOR

DISPLAY "I-Isc succesfully calculated";

DISPLAY "Next, CoC is built";

ALLOCATEMEMORY to CC WITH num BYTES;

STARTIF CC = NULL

THEN

DISPLAY "Not enough memory, the program exits now, press any key";

GETCHARACTER;

ENDIF;

ALLOCATE CC[0]=IminIsc[0];

ALLOCATE CC[1]=deltax*(IminIsc[1]+IminIsc[0])/2;

ALLOCATE CC[2]=CC[0]+(deltax/3)*(IminIsc[2]+4*IminIsc[1]+IminIsc[0]);

ALLOCATE CC[3]=CC[0]+(3*deltax/8)*(IminIsc[3]+3*IminIsc[2]+3*IminIsc[1]+IminIsc[0]);

ALLOCATE CC[4]=CC[0]+(2*deltax/45)*(7*IminIsc[4]+32*IminIsc[3]+12*IminIsc[2] +32*IminIsc[1]+7*IminIsc[0]);

STARTFOR i=5 DOWHILE i<num AND ALLOCATE i = i+1

ALLOCATE CC[i]=CC[i-5]+(5*deltax/288)*(19*IminIsc[i-5]+75*IminIsc[i-4]+50*IminIsc[i-3]+50*IminIsc[i-2]+75*IminIsc[i-1]+19*IminIsc[i]);

ENDFOR

DISPLAY "CC succesfully calculated";

STARTFOR i=0 DOWHILE i<num AND ALLOCATE i = i+1

ALLOCATE sumx = sumx + xdata[i];

ALLOCATE sumx2 = sumx2 + xdata[i]*xdata[i];

ALLOCATE sumx3 = sumx3 + xdata[i]*xdata[i]*xdata[i];

ALLOCATE sumx4 = sumx4 + xdata[i]*xdata[i]*xdata[i]*xdata[i];

ALLOCATE sumy = sumy + IminIsc[i];

ALLOCATE sumy2 = sumy2 + IminIsc[i]*IminIsc[i];

ALLOCATE sumy3 = sumy3 + IminIsc[i]*IminIsc[i]*IminIsc[i];

ALLOCATE sumy4 = sumy4 + IminIsc[i]*IminIsc[i]*IminIsc[i]*IminIsc[i];

ALLOCATE sumxy = sumxy + xdata[i]*IminIsc[i];

ALLOCATE sumxy2 = sumxy2 + xdata[i]*IminIsc[i]*IminIsc[i];

ALLOCATE sumx2y = sumx2y + xdata[i]*xdata[i]*IminIsc[i];

ALLOCATE sumx2y2 = sumx2y2 + xdata[i]*xdata[i]*IminIsc[i]*IminIsc[i];

ALLOCATE sumx3y = sumx3y + xdata[i]*xdata[i]*xdata[i]*IminIsc[i];

ALLOCATE sumxy3 = sumxy3 + xdata[i]*IminIsc[i]*IminIsc[i]*IminIsc[i];

ENDFOR

DISPLAY "Now, vector V is calculated";

ALLOCATE v[0] = v[1] = v[2] = v[3] = v[4] = v[5] = 0;

STARTFOR i=0 DOWHILE i<num AND ALLOCATE i = i+1

ALLOCATE sumz = sumz + CC[i];

ALLOCATE sumxz = sumxz + xdata[i]*CC[i];

ALLOCATE sumx2z = sumx2z + xdata[i]*xdata[i]*CC[i];

ALLOCATE sumyz = sumyz + IminIsc[i]*CC[i];

ALLOCATE sumy2z = sumy2z + IminIsc[i]*IminIsc[i]*CC[i];

ALLOCATE sumxyz = sumxyz + xdata[i]*IminIsc[i]*CC[i];

ENDFOR

ALLOCATE v[0] = sumz;

ALLOCATE v[1] = sumxz;

ALLOCATE v[2] = sumx2z;

ALLOCATE v[3] = sumyz;

ALLOCATE v[4] = sumy2z;

ALLOCATE v[5] = sumxyz;

DISPLAY "V succesfully calculated";

DISPLAY "The vector V is";

DISPLAY "Sz = %0.3f ENDOFLINE Sxz = %0.3f ENDOFLINE Sx2z = %0.3f ENDOFLINE Syz = %0.3f ENDOFLINE Sy2z = %0.3f ENDOFLINE Sxyz = %0.3f ENDOFLINE", sumz, sumxz, sumx2z, sumyz, sumy2z, sumxyz;

DISPLAY "Sum CoCi = %0.3f ENDOFLINE Sum ViCoCi = %0.3f ENDOFLINE Sum Vi^2CoCi = %0.3f ENDOFLINE Sum (I-Isc)iCoCi = %0.3f ENDOFLINE Sum ((I-Isc)i^2)CoCi= %0.3f ENDOFLINE Sum Vi(I-Isc)iCoCi = %0.3f ENDOFLINE ", sumz, sumxz, sumx2z, sumyz, sumy2z, sumxyz);

DISPLAY "The matrix Mat = WtW, is DOnow";

ALLOCATE maux[0][0]=num;

ALLOCATE maux[0][1]=sumx;

ALLOCATE maux[0][2]=sumx2;

ALLOCATE maux[0][3]=sumy;

ALLOCATE maux[0][4]=sumy2;

ALLOCATE maux[0][5]=sumxy;

ALLOCATE maux[1][0]=sumx;

ALLOCATE maux[1][1]=sumx2;

ALLOCATE maux[1][2]=sumx3;

ALLOCATE maux[1][3]=sumxy;

ALLOCATE maux[1][4]=sumxy2;

ALLOCATE maux[1][5]=sumx2y;

ALLOCATE maux[2][0]=sumx2;

ALLOCATE maux[2][1]=sumx3;

ALLOCATE maux[2][2]=sumx4;

ALLOCATE maux[2][3]=sumx2y;

ALLOCATE maux[2][4]=sumx2y2;

ALLOCATE maux[2][5]=sumx3y;

ALLOCATE maux[3][0]=sumy;

ALLOCATE maux[3][1]=sumxy;

ALLOCATE maux[3][2]=sumx2y;

ALLOCATE maux[3][3]=sumy2;

ALLOCATE maux[3][4]=sumy3;

ALLOCATE maux[3][5]=sumxy2;

ALLOCATE maux[4][0]=sumy2;

ALLOCATE maux[4][1]=sumxy2;

ALLOCATE maux[4][2]=sumx2y2;

ALLOCATE maux[4][3]=sumy3;

ALLOCATE maux[4][4]=sumy4;

ALLOCATE maux[4][5]=sumxy3;

ALLOCATE maux[5][0]=sumxy;

ALLOCATE maux[5][1]=sumx2y;

ALLOCATE maux[5][2]=sumx3y;

ALLOCATE maux[5][3]=sumxy2;

ALLOCATE maux[5][4]=sumxy3;

ALLOCATE maux[5][5]=sumx2y2;

DISPLAY "Matrix Mat = WtW succesfully calculated";

DISPLAY "Now, the inverse of Mat = WtW, namely (WtW)^-1 or inv(WtW), is calculated";

CALCULATEINVERSEMATRIX maux[][];

DISPLAY "Matrix (WtW)^-1 or inv(WtW) succesfully calculated";

DISPLAY "The matrix W is built next";

STARTFOR i4=0 DOWHILE i<num AND ALLOCATE i4 = i4+1

ALLOCATE auxX[6*i4 + 0] = 1;

ALLOCATE auxX[6*i4 + 1] = xdata[i4];

ALLOCATE auxX[6*i4 + 2] = xdata[i4]^2;

ALLOCATE auxX[6*i4 + 3] = IminIsc[i4];

ALLOCATE auxX[6*i4 + 4] = IminIsc[i4]^2;

ALLOCATE auxX[6*i4 + 5] = xdata[i4]*IminIsc[i4];

ENDFOR

DISPLAY "Matrix W succesfully calculated";

DISPLAY "The transpose of W, Wt, is built now";

STARTFOR i4=0 DOWHILE i<num AND ALLOCATE i4 = i4+1

ALLOCATE auxtranspX[num*0 + i4] = 1;

ALLOCATE auxtranspX[num*1 + i4] = xdata[i4];

ALLOCATE auxtranspX[num*2 + i4] = xdata[i4]^2;

ALLOCATE auxtranspX[num*3 + i4] = IminIsc[i4];

ALLOCATE auxtranspX[num*4 + i4] = IminIsc[i4]^2;

ALLOCATE auxtranspX[num*5 + i4] = xdata[i4]*IminIsc[i4];

ENDFOR

DISPLAY "Matrix Wt succesfully calculated";

DISPLAY "The product (WtW)^-1Wt is DOne next";

STARTFOR i1=0 DOWHILE i1<6 AND ALLOCATE i1 = i1+1

STARTFOR i2=0 DOWHILE i2<num AND ALLOCATE i2 = i2+1

STARTFOR i3=0 DOWHILE i3<6 AND ALLOCATE i3 = i3+1

ALLOCATE auxA[num*i1 + i2] = auxA[num*i1 + i2] + mident[i1][i3]*auxtranspX[num*i3 + i2];

ENDFOR

ENDFOR

ENDFOR

DISPLAY "The product (WtW)^-1Wt succesfully calculated";

DISPLAY "Next, the Hat matrix, Hat, is calculated as Hat=W(WtW)^-1Wt";

DISPLAY "Also, the Identity - Hat, i.e., 1-Hat, is calculated";

STARTFOR i1=0 DOWHILE i1<num AND ALLOCATE i1 = i1+1

STARTFOR i2=0 DOWHILE i2<num AND ALLOCATE i2 = i2+1

STARTFOR i3=0 DOWHILE i3<6 AND ALLOCATE i3 = i3+1

ALLOCATE Hat[num*i1 + i2] = Hat[num*i1 + i2] + auxX[6*i1 + i3]*auxA[num*i3 + i2];

ENDFOR

STARTIF i1=i2

THEN

IminHat[num*i1 + i2] = 1 - Hat[num*i1 + i2];

ELSE

IminHat[num*i1 + i2] = -Hat[num*i1 + i2];

ENDIF

ENDFOR

ENDFOR

ALLOCATE traceH = 0;

STARTFOR i1=0 DOWHILE i1<num AND ALLOCATE i1 = i1+1

traceH = traceH + Hat[num*i1 + i1];

ENDFOR

DISPLAY "Matrix Hat succesfully calculated";

DISPLAY "Matrix 1-Hat succesfully calculated";

DISPLAY "The trace of Hat = %E", traceH;

DISPLAY "The vector E is built now as E = (1-Hat)CoC";

STARTFOR i1=0 DOWHILE i1<num AND ALLOCATE i1 = i1+1

aux1 = 0;

STARTFOR i2=0 DOWHILE i2<num AND ALLOCATE i2 = i2+1

aux1 = aux1 + IminHat[num*i1 + i2]*CC[i2];

ENDFOR

auxE[i1] = aux1;

ENDFOR

ALLOCATE Sumae2 = 0;

STARTFOR i1=0 DOWHILE i1<num AND ALLOCATE i1 = i1+1

Sumae2 = Sumae2 + auxE[i1]^2;

ENDFOR

DISPLAY "Vector E succesfully calculated";

DISPLAY "The Sume E^2 = %E", Sumae2;

ALLOCATE Sig2 = Sumae2/(num-traceH);

DISPLAY "The value of Sigma = %E", Sig2;

DISPLAY "The covariance matrix is built now as Sigma*((WtW)^-1)";

STARTFOR i1=0 DOWHILE i1<6 AND ALLOCATE i1 = i1+1

STARTFOR i2=0 DOWHILE i2<6 AND ALLOCATE i2 = i2+1

covariance[i1][i2] = Sig2*mident[i1][i2];

ENDFOR

ENDFOR

DISPLAY "Covariance matrix succesfully calculated";

ALLOCATE errCv0 = dstda = covariance[0][0]^0.5;

ALLOCATE errCv1 = dstdb = covariance[1][1]^0.5;

ALLOCATE errCv2 = dstdc = covariance[2][2]^0.5;

ALLOCATE errCi1 = dstdd = covariance[3][3]^0.5;

ALLOCATE errCi2 = dstde = covariance[4][4]^0.5;

ALLOCATE errCv1i1 = dstdf = covariance[5][5]^0.5;

SOLVEMATRIXEQUATION STARTFOR MATRIX maux AND vector V TOOBTAIN Cv0 AND Cv1 AND Cv2 AND Ci1 AND Ci2 AND Cv1i1;

DISPLAY "In Summary, the Cs + deltaCs values are";

DISPLAY "Cv0 +- errCv0 = %E +- %E", Cv0, errCv0;

DISPLAY "Cv1 +- errCv1 = %E +- %E", Cv1, errCv1;

DISPLAY "Cv2 +- errCv2 = %E +- %E", Cv2, errCv2;

DISPLAY "Ci1 +- errCi1 = %E +- %E", Ci1, errCi1;

DISPLAY "Ci2 +- errCi2 = %E +- %E", Ci2, errCi2;

DISPLAY "Cv1i1 +- errCv1i1 = %E +- %E\n", Cv1i1, errCv1i1;

ALLOCATE A = (1+16*Ci2*Cv2)^0.5;

ALLOCATE Rsh = 1/(2*Cv2);

ALLOCATE errRsh = -errCv2/(2*(Cv2^2));

ALLOCATE Rs = (A-1)/(4*Cv2);

ALLOCATE errRs = (0.5)*(((A-(1+8*Cv2*Ci2))/(A*(Cv2^2)))*errCv2 + (8*errCi2)/(A));

ALLOCATE n = (Cv1*(A-1)+4*Ci1*Cv2)/(4*Vth*Cv2);

ALLOCATE errn = ((A-1)/(4*Vth*Cv2))*errCv1 +

errCv2*(Cv1*(A-(1+8*Cv2*Ci2)))/(4*Vth*A*Cv2) +

errCi1/Vth +

errCi2*(2*Cv1)/(A*Vth);

ALLOCATE Ilig= - (1+A)*(Cv1+Isc)/2 - 2*Ci1*Cv2;

ALLOCATE errIlig = - ((1+A)/(2))*errCv1 -

((4*Ci2*(Cv1+Isc))/(A) + 2*Ci1)*errCv2 -

2*Cv2*errCi1 -

errCi2*(4*(Cv1 + Isc)*Cv2)/(A);

DISPLAY "The five solar cell parameters are: ";

DISPLAY "Rs +- errRs = %E +- %E", Rs, ABS(errRs);

DISPLAY "Rsh +- errRsh = %E +- %E", Rsh, ABS(errRsh);

DISPLAY "n +- errn = %E +- %E", n, ABS(errn);

DISPLAY "Ilig +- errIlig = %E +- %E", Ilig, ABS(errIlig);

DISPLAY "Now absI absIminIsc B Isat absIsat errIsat abserrIsat are built";

ALLOCATEMEMORY to absI WITH num BYTES;

STARTIF absI = NULL

THEN

DISPLAY "Not enough memory, the program exits now, press any key";

GETCHARACTER;

ENDIF;

ALLOCATEMEMORY to absIminIsc WITH num BYTES;

STARTIF absIminIsc = NULL

THEN

DISPLAY "Not enough memory, the program exits now, press any key";

GETCHARACTER;

ENDIF;

ALLOCATEMEMORY to B WITH num BYTES;

STARTIF B = NULL

THEN

DISPLAY "Not enough memory, the program exits now, press any key";

GETCHARACTER;

ENDIF;

ALLOCATEMEMORY to Isat WITH num BYTES;

STARTIF Isat = NULL

THEN

DISPLAY "Not enough memory, the program exits now, press any key";

GETCHARACTER;

ENDIF;

ALLOCATEMEMORY to absIsat WITH num BYTES;

STARTIF absIsat = NULL

THEN

DISPLAY "Not enough memory, the program exits now, press any key";

GETCHARACTER;

ENDIF;

ALLOCATEMEMORY to errIsat WITH num BYTES;

STARTIF errIsat = NULL

THEN

DISPLAY "Not enough memory, the program exits now, press any key";

GETCHARACTER;

ENDIF;

ALLOCATEMEMORY to abserrIsat WITH num BYTES;

STARTIF abserrIsat = NULL

THEN

DISPLAY "Not enough memory, the program exits now, press any key";

GETCHARACTER;

ENDIF;

STARTFOR i1=0 DOWHILE i1<num AND ALLOCATE i1 = i1+1

ALLOCATE absI[i1] = ABS(ydata[i1]);

ALLOCATE absIminIsc[i1] = ABS(IminIsc[i1]);

ALLOCATE B[i1] = 1/(exp((xdata[i1]-ydata[i1]*Rs)/(n*Vth))-1);

ALLOCATE Isat[i1]= B[i1]*(ydata[i1] - Ilig - (xdata[i1]-ydata[i1]*Rs)/(Rsh));

absIsat[i1] = ABS(Isat[i1]);

ALLOCATE errIsat[i1] = errI*B[i1]*(1 + Rs/Rsh - (Rs/(n*Vth))*(B[i1]+1)*(ydata[i1] + Ilig - (xdata[i1]-ydata[i1]*Rs)/(Rsh))) + B[i1]*errIlig + B[i1]*ydata[i1]*(1/Rs - ((B[i1]+1)/(n*Vth))*(ydata[i1]+Ilig-(xdata[i1]-ydata[i1]*Rs)/(Rsh)))*errRs + B[i1]*((xdata[i1]-ydata[i1]*Rs)/(Rsh*Rsh))*errRsh - B[i1]*(B[i1]+1)*((ydata[i1]*Rs)/(n*n*Vth))*(ydata[i1]+Ilig-(xdata[i1]- ydata[i1]*Rs)/(Rsh))*errn;

abserrIsat[i1] = ABS(errIsat[i1]);

ENDFOR

IsatMaxV = Isat[num-1];

errIsatMaxV = errIsat[num-1];

DISPLAY "The value of Isat at maximum voltage is = %E\n", IsatMaxV;

DISPLAY "The value of errIsat at maximum voltage is = %E\n", errIsatMaxV;

STARTIF safematrixes = 'y' OR safematrixes = 'Y'

THEN

DISPLAY "You mentioned at the beginning of the program to WRITEINFILE W, Wt, Mat = WtW, inv(WtWY), Hat, 1-Hat, E, CoC, Covariance, and V matrixes, this is DOnext";

DISPLAY "CoC is saved next, in CoC.txt";

WRITEINFILE CC[] INFILE "CoC.txt";

DISPLAY "Data CoC written to CoC.txt successfully."

DISPLAY "The V vector is saved now as V.txt";

WRITEINFILE V[] INFILE "V.txt";

DISPLAY "Data V written to V.txt successfully."

DISPLAY "\nThe Mat = WtW is saved now as WtW.txt";

WRITEINFILE maux[] INFILE "WtW.txt";

DISPLAY "Data Mat = WtW written to WtW.txt successfully."

DISPLAY "The (WtW)^-1 is saved now as inv(WtW).txt";

WRITEINFILE maux[] INFILE " inv(WtW).txt";

DISPLAY "Data (WtW)^-1 written to inv(YtY).txt successfully.”;

DISPLAY "The W is saved now as W.txt";

WRITEINFILE auxX[] INFILE "W.txt ";

DISPLAY "Data W written to W.txt successfully."

DISPLAY "The Wt is saved now as Wt.txt";

WRITEINFILE auxtranspX [] INFILE "Wt.txt";

DISPLAY "Data Wt written to Wt.txt successfully."

DISPLAY "The Hat matrix is saved now as Hat.txt";

WRITEINFILE Hat[] INFILE "Hat.txt";

DISPLAY "Data Hat H written to Hat.txt successfully."

DISPLAY "The 1-Hat = 1-Hat matrix, is saved now as 1-Hat.txt";

WRITEINFILE IminHat[] INFILE "1-Hat.txt";

DISPLAY "Data 1-Hat written to 1-Hat.txt successfully."

DISPLAY "The E vector is saved now as E.txt";

WRITEINFILE auxE[] INFILE "E.txt";

DISPLAY "Data E written to E.txt successfully."

DISPLAY "The Covariance matrix is saved now as Cov.txt";

WRITEINFILE covariance[] INFILE "Cov.txt";

DISPLAY “Data Covariance written to Cov.txt successfully.";

GETTIME_t time2;

DISPLAY "The total computation time was %d", time2 - time1;

DISPLAY "Now Results1, Results2, and V-I-absI-IminIsc-absIminIsc-CoC-B-Isat-absIsat-errIsat-abserrIsat are saved";

WRITEINFILE "Cv0 ENDOFLINE deltaCv0 ENDOFLINE Cv1 ENDOFLINE deltaCv1 ENDOFLINE Cv2 ENDOFLINE deltaCv2 ENDOFLINE Ci1 ENDOFLINE deltaCi1 ENDOFLINE Ci2 ENDOFLINE deltaCi2 ENDOFLINE Cv1i1 ENDOFLINE deltaCv1i1" INFILE "Results1.txt";

WRITEINFILE Cv0 , errCv0 , Cv1 , errCv1 , Cv2 , errCv2 , Ci1 , errCi1 , Ci2 , errCi2 , Cv1i1 , errCv1i1 INFILE "Results1.txt";

WRITEINFILE "Rs (ohms) ENDOFLINE errRs (ohms) ENDOFLINE Rsh (ohms) ENDOFLINE errRsh (ohms) ENDOFLINE errn ENDOFLINE Ilig (A) ENDOFLINE errIlig (A) ENDOFLINE IsatMaxV (A) ENDOFLINE errIsatMaxV (A)" INFILE "Results1.txt";

WRITEINFILE Rs , errRs , Rsh , errRsh , n , errn , Ilig , errIlig , IsatMaxV , errIsatMaxV INFILE "Results1.txt";

WRITEINFILE "Total number of pair of data = " INFILE "Results1.txt";

WRITEINFILE "Total computational time = " time2 - time1 " seconds " INFILE "Results1.txt";

WRITEINFILE "Remember to simulate your IV curves with the deduced parameters, and to compare to your experimental IV, to ensure good solar cell parameter extraction" INFILE "Results1.txt";

WRITEINFILE "If this program has helped you in your research and producing your articles, it will be strongly appreciated if you can cite the article, to give it diffusion in the research community" INFILE "Results1.txt";

WRITEINFILE "so more researchers and research groups will be benefited from it. The article citation information is\n" INFILE "Results1.txt";

WRITEINFILE " Five solar cell parameters automatic extraction, within the one diode-solar cell model,\nusing the implemented Simpson Order 5 integration method, in an executable program. Victor-Tapio Rangel-Kuoppa " INFILE "Results3.txt";

WRITEINFILE " Submitted to PLoS One (2025)" INFILE "Results3.txt";

WRITEINFILE "\nRemember to read the following articles, to see how to reduce the influence of noise, increasing the number of points, using the trapezoidal integration\n " INFILE "Results1.txt";

WRITEINFILE "DOI 10.1088/2631-8695/ac4c36" INFILE "Results1.txt";

WRITEINFILE "DOI 10.1088/2631-8695/ac4c37" INFILE "Results1.txt";

WRITEINFILE "DOI 10.1088/2631-8695/ace759" INFILE "Results1.txt";

WRITEINFILE "DOI 10.1088/2631-8695/ace590" INFILE "Results1.txt";

DISPLAY "Data written to Results1.txt successfully."

WRITEINFILE ""Cv0 ± deltaCV0 TAB Cv1 ± deltaCV1 TAB Cv2 ± deltaCV2 ENDOFLINE Ci1 ± deltaCi1 TAB Ci2 ± deltaCi2 TAB Cv1i1 ± deltaCv1i1" INFILE "Results2.txt";

WRITEINFILE Cv0 " ± " errCv0 TAB Cv1 " ± " errCv1 TAB Cv2 " ± " errCv2 TAB Ci1 " ± " errCi1 TAB Ci2 " ± " errCi2 TAB Cv1i1 " ± " errCv1i1 INFILE "Results2.txt";

WRITEINFILE Rs , errRs , Rsh , errRsh , n , errn , Ilig , errIlig , IsatMaxV , errIsatMaxV INFILE "Results2.txt";

WRITEINFILE "Rs ± deltaRs (ohms)" TAB "Rsh ± deltaRsh (ohms)" TAB "n ± deltan" TAB "Ilig ± deltaIlig (A)" TAB "IsatMaxV ± deltaIsatMaxV (A)" INFILE "Results2.txt";

WRITEINFILE Rs " ± " ABS(errRs) TAB Rsh << " ± " << ABS (errRsh) TAB n " ± " ABS (errn) TAB Ilig " ± " ABS (errIlig) TAB IsatMaxV " ± " ABS (errIsatMaxV) INFILE "Results2.txt";

WRITEINFILE "Total number of pair of data = " INFILE "Results2.txt";

WRITEINFILE "Total computational time = " time2 - time1 " seconds " INFILE "Results2.txt"; WRITEINFILE "Remember to simulate your IV curves with the deduced parameters, and to compare to your experimental IV, to ensure good solar cell parameter extraction" INFILE "Results2.txt";

WRITEINFILE "If this program has helped you in your research and producing your articles, it will be strongly appreciated If you can cite the article, to give it diffusion in the research community" INFILE "Results2.txt";

WRITEINFILE " Five solar cell parameters automatic extraction, within the one diode-solar cell model,\nusing the implemented Simpson Order 5 integration method, in an executable program. Victor-Tapio Rangel-Kuoppa " INFILE "Results3.txt";

WRITEINFILE " Submitted to PLoS One (2025)" INFILE "Results3.txt";

WRITEINFILE "\nRemember to read the following articles, to see how to reduce the influence of noise, increasing the number of points, using the trapezoidal integration\n " INFILE "Results2.txt";

WRITEINFILE "DOI 10.1088/2631-8695/ac4c36" INFILE "Results2.txt";

WRITEINFILE "DOI 10.1088/2631-8695/ac4c37" INFILE "Results2.txt";

WRITEINFILE "DOI 10.1088/2631-8695/ace759" INFILE "Results2.txt";

WRITEINFILE "DOI 10.1088/2631-8695/ace590" INFILE "Results2.txt";

DISPLAY "Data written to Results1.txt successfully."

WRITEINFILE "V-I-absI-IminIsc-absIminIsc-CoC-B-Isat-absIsat-errIsat-abserrIsat.txt" INFILE "Results3.txt";

WRITEINFILE "V" TAB "I" TAB "absI" TAB "IminIsc" TAB "absIminIsc" TAB "CoC" TAB "B" TAB "Isat" TAB "absIsat" TAB "deltaIsat" TAB "absdeltaIsat" INFILE "Results3.txt";

WRITEINFILE xdata[] TAB ydata[] TAB absI[] TAB IminIsc[] TAB absIminIsc[] TAB CC[] TAB B[] TAB Isat[] TAB absIsat[] TAB errIsat[] TAB abserrIsat[] INFILE "Results3.txt";

WRITEINFILE "Total number of pair of data = " INFILE "Results3.txt";

WRITEINFILE "Total computational time = " time2 - time1 " seconds " INFILE "Results3.txt";

WRITEINFILE "Remember to simulate your IV curves with the deduced parameters, and to compare to your experimental IV, to ensure good solar cell parameter extraction" INFILE "Results3.txt";

WRITEINFILE "If this program has helped you in your research and producing your articles, it will be strongly appreciated if you can cite the article, to give it diffusion in the research community" INFILE "Results3.txt";

WRITEINFILE "so more researchers and research groups will be benefited from it. The article citation information is\n" INFILE "Results3.txt";

WRITEINFILE " Five solar cell parameters automatic extraction, within the one diode-solar cell model,\nusing the implemented Simpson Order 5 integration method, in an executable program. Victor-Tapio Rangel-Kuoppa " INFILE "Results3.txt";

WRITEINFILE " Submitted to PLoS One (2025)" INFILE "Results3.txt";

WRITEINFILE "\nRemember to read the following articles, to see how to reduce the influence of noise, increasing the number of points, using the trapezoidal integration\n " INFILE "Results3.txt";

WRITEINFILE "DOI 10.1088/2631-8695/ac4c36" INFILE "Results3.txt";

WRITEINFILE "DOI 10.1088/2631-8695/ac4c37" INFILE "Results3.txt";

WRITEINFILE "DOI 10.1088/2631-8695/ace759" INFILE "Results3.txt";

WRITEINFILE "DOI 10.1088/2631-8695/ace590" INFILE "Results3.txt";

DISPLAY "Data written on Results3.txt successfully."

ENDIF

DISPLAY "PRESS ANY KEY TO END THE PROGRAM";

GETCHARACTER;

FREEMEMORY xdata;

FREEMEMORY ydata;

FREEMEMORY helpxdata;

FREEMEMORY helpydata;

FREEMEMORY IminIsc;

FREEMEMORY CC;

FREEMEMORY absI;

FREEMEMORY absIminIsc;

FREEMEMORY B;

FREEMEMORY Isat;

FREEMEMORY absIsat;

FREEMEMORY errIsat;

FREEMEMORY abserrIsat;

FREEMEMORY auxX;

FREEMEMORY auxtranspX;

FREEMEMORY auxA;

FREEMEMORY Hat;

FREEMEMORY IminHat;

FREEMEMORY auxE;

ENDPROGRAM;
