# Supplementary figures and images for: Five solar cell parameters automatic extraction, within the one diode-solar cell model, using the implemented Simpson order 5 integration method, in an executable program
Source: PLoS One. 2026 Apr 22;21(4):e0346051. doi: 10.1371/journal.pone.0346051 (PMC13102238; doi:10.1371/journal.pone.0346051)

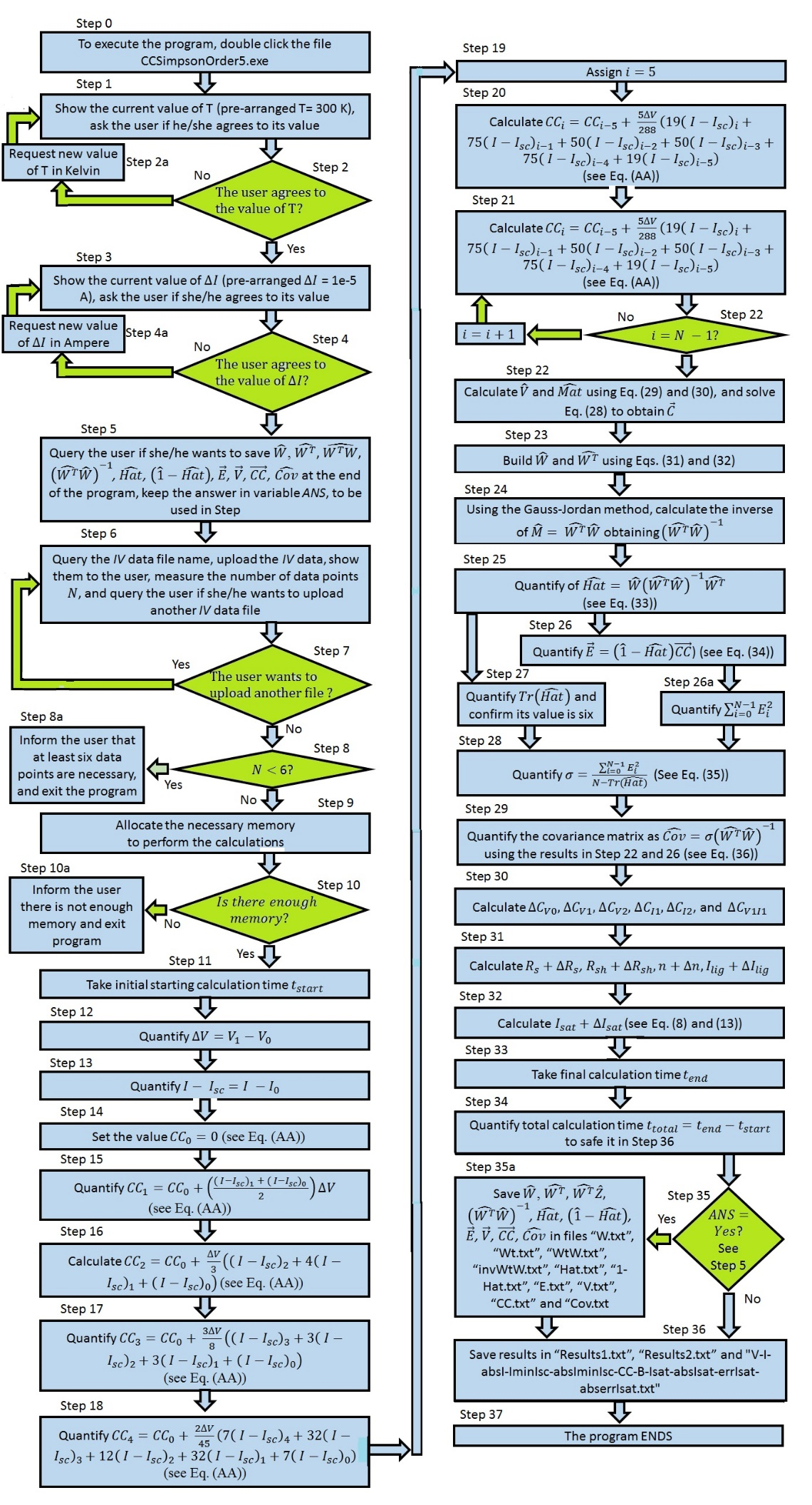

Supplement: S1 Fig — (BMP) [file pone.0346051.s003.bmp]

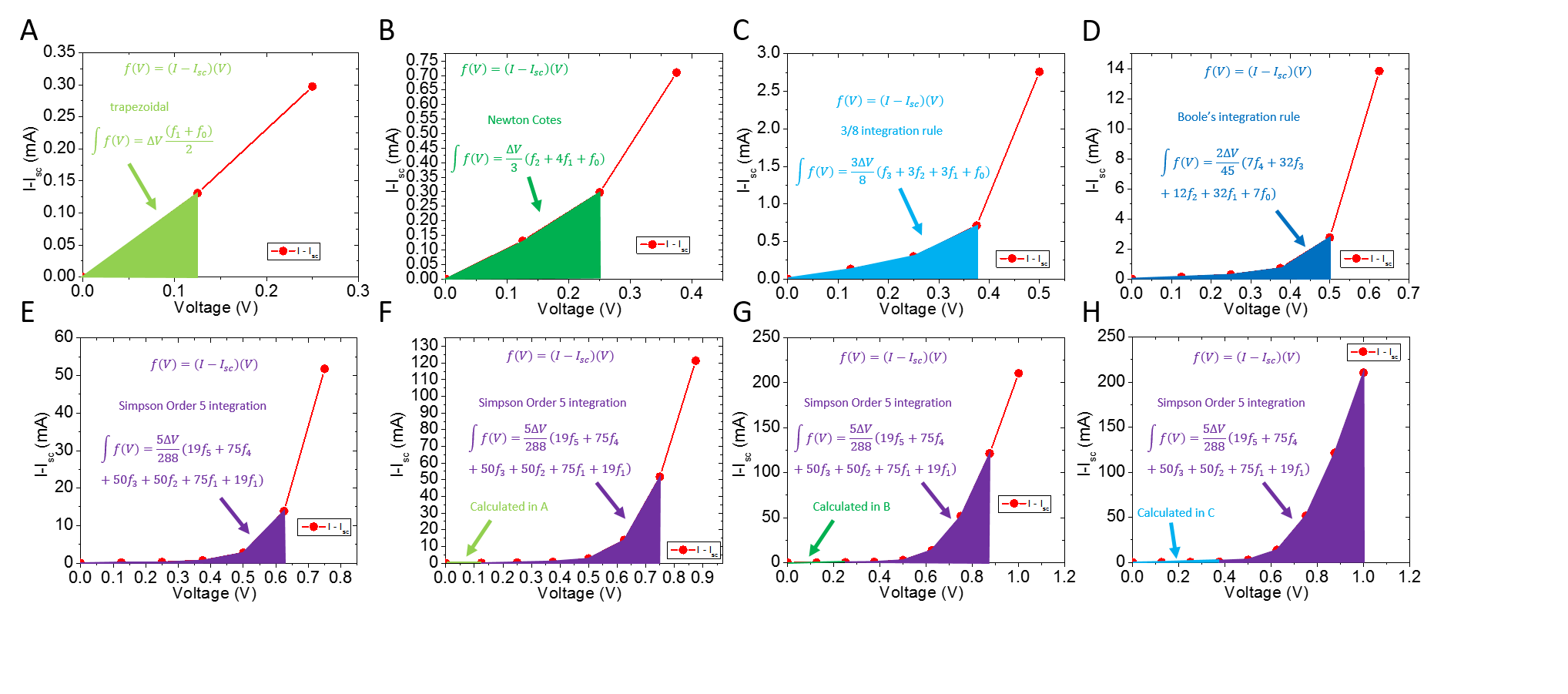

Supplement: S2 Fig — Visual explanation, on how the 𝐂𝐂(𝐕,𝐈) is calculated in S1 Table, in A using the trapezoidal integration, in B the Newton Cotes integration, in C, the 3/8 integration, in D the Boole’s integration, in E the order 5 Simpson integration, F the order 5 Simpson integration, in F the order 5 Simpson integration, adding it to the integration obtained in A, in G the order 5 Simpson integration, adding it to the integration obtained in B, and in H the order 5 Simpson integration, adding it to the integration obtained in C. (TIF) [file pone.0346051.s004.tif]
