# Supplementary material for: Five solar cell parameters automatic extraction, within the one diode-solar cell model, using the implemented Simpson order 5 integration method, in an executable program
Source: PLoS One. 2026 Apr 22;21(4):e0346051. doi: 10.1371/journal.pone.0346051 (PMC13102238; doi:10.1371/journal.pone.0346051)
Supplement: S1 Table — First, in Column 1 and Column 2, namely Col1 and Col2, the IV data from file I-V9points.txt, is given. Afterwards, in Col3, I – Isc, is reported, as the value of Column 2, minus the value (Line 1, Column 2), i.e., Col2 – (L1, Col2). Next, in Col4, an explanation on how CC(V, I) was calculated is given, using the trapezoidal integration method (Eq. (15)) in L2, the Newton Cotes integration (Eq. (16)) method in L3, the 3/8 rule (Eq. (17)) in L4, and then, from L5 to L8, the Boole’s integration formula (Eq. (18)), adding it to value in the previous line and same column is shown. For the sake of clarity, the numerical calculations are given. Finally, in Col5 the final value of CC(V, I) is reported. (DOCX) [file pone.0346051.s005.docx]

**S1 Table**. Example on the application of program CCSimpsonOrder5.exe, on *IV* data file I-V9points.txt, available in the Supplementary Material. First, in Column 1 and Column 2, namely Col1 and Col2, the *IV* data from file I-V9points.txt, is given. Afterwards, in Col3, *I - I_sc_*, is reported, as the value of Column 2, minus the value (Line 1, Column 2), i.e., Col2 – (L1, Col2). Next, in Col4, an explanation on how *CC(V, I)* was calculated is given, using the trapezoidal integration method (Eq. (15)) in L2, the Newton Cotes integration (Eq. (16)) method in L3, the 3/8 rule (Eq. (17)) in L4, and then, from L5 to L8, the Boole’s integration formula (Eq. (18)), adding it to value in the previous line and same column is shown. For the sake of clarity, the numerical calculations are given. Finally, in Col5 the final value of *CC(V, I)* is reported.

| Line / Column | Col1 | Col2 | Col3 | Col4 | Col5 |
| --- | --- | --- | --- | --- | --- |
|  | Voltage  *V* (V) | Current  *I* (mA) | $f\left( V \right)=I-I_{sc}$(mA)  Col2 – (L1, Col2) | Explanation of which integration was used  to calculate *CC(V, I)* | *CC(V, I)*  *(mW)* |
| L1 | 0 | -0.999 | 0 | $\int_{0 V}^{0 V} f\left( V \right)=0 mW$ | 0 m*W* |
| L2 | 0.125 | -0.868 | 0.1309 | Trapezoidal integration is used in this case, adding it to the result in $\left( L1, Col5 \right)$, see Fig S2A  $\int_{0 V}^{0.125 V} f\left( V \right)\approx\left( L1, Col5 \right)+\Delta V\left( \frac{f\left( 0 V \right)+f\left( 0. 125 V \right)}{2} \right)=0 mW+\left( 0.125 V \right)\left( \frac{0 mA+0.1309 mA}{2} \right)=0.00818 mW$ | 0.00818 m*W* |
| L3 | 0.25 | -0.702 | 0.2974 | Newton Cotes integration is used in this case, adding it to the result in $\left( L1, Col5 \right)$, see Fig S2B  $\int_{0 V}^{0.25 V} f\left( V \right)\approx\left( L1, Col5 \right)+\left( \frac{\Delta V}{3} \right)\left( f\left( 0.25 V \right)+4f\left( 0. 125 V \right)+f\left( 0 V \right) \right)=0 mW+\left( \frac{0.125 V}{3} \right)\left( 0.2974 mA+4\left( 0.1309 mA \right)+0 mA \right)=0.03421 mW$ | $0.03421$ *mW* |
| L4 | 0.375 | -0.289 | 0.7097 | 3/8 integration is used in this case, adding it to the result in $\left( L1, Col5 \right)$, see Fig S2C  $\int_{0 V}^{0.375 V} f\left( V \right)\approx\left( L1, Col5 \right)+\left( \frac{3\Delta V}{8} \right)\left( f\left( 0.375 V \right)+3f\left( 0.25 V \right)++3f\left( 0.125 V \right)+f\left( 0 V \right) \right)= 0 mW+\left( \frac{3\times0.125 V}{8} \right)\left( 0.7097 mA+3\left( 0.2974 mA \right)+3\left( 0.1309 mA \right)+0 mA \right)=0.09349 mW$ | $0.09349$ *mW* |
| L5 | 0.5 | 1.761 | 2.7596 | Boole´s integration formula is used in this case, adding it to the result in $\left( L1, Col5 \right)$, see Fig S2D  $\int_{0 V}^{0.5 V} f\left( V \right)\approx\left( L1, Col5 \right)+\frac{2\Delta V}{45}\left( 7f\left( 0.5 V \right)+32f\left( 0.375 V \right)+12f\left( 0.25 V \right)+32f\left( 0.125 V \right)+7f\left( 0 V \right) \right)=0 mW+\frac{2\left( 0.125 V \right)}{45}\left( 7\times2.7596 mA+32\times0.7097 mA+12\times0.2974 mA+32\times0.1309 mA+7\times0 mA \right)=0.27658 mW$ | $0.27658 mW$ |
| L6 | 0.625 | 12.847 | 13.8466 | Order 5 Simpson integration formula is used in this case, adding it to the result in $\left( L1, Col5 \right)$, see Fig S2E  $\int_{0 V}^{0.625 V} f\left( V \right)\approx\left( L1, Col5 \right)+\frac{5\Delta V}{288}\left( 19f\left( 0.625 V \right)+75f\left( 0.5 V \right)+50f\left( 0.375 V \right)+50f\left( 0.25 V \right)+75f\left( 0.125 V \right)+19f\left( 0 V \right) \right)=0 mW+\frac{5\left( 0.125 V \right)}{288}\left( 19\times13.8466 mA+75\times2.7596 mA+50\times0.7097 mA+50\times0.2974 mA+75\times0.1309 mA+19\times0 mA \right)=1.15069 mW$ | $1.15069$*mW* |
| L7 | 0.75 | 50.763 | 51.7629 | Order 5 Simpson integration formula is used in this case, adding it to the result in $\left( L2, Col5 \right)$, see Fig S2F  $\int_{0 V}^{0.75 V} f\left( V \right)\approx\left( L2, Col5 \right)+\frac{5\Delta V}{288}\left( 19f\left( 0.75 V \right)+75f\left( 0.625 V \right)+50f\left( 0.5 V \right)+50f\left( 0.375 V \right)+75f\left( 0.25 V \right)+19f\left( 0.125 V \right) \right)=0.00818 mW+\frac{5\left( 0.125 V \right)}{288}\left( 19\times51.7629 mA+75\times13.8466 mA+50\times2.7596 mA+50\times0.7097 mA+75\times0.2974 mA+19\times0.1309 mA \right)=4.82645 mW$ | $4.82645 mW$ |
| L8 | 0.875 | 120.341 | 121.34 | Order 5 Simpson integration formula is used in this case, adding it to the result in $\left( L3, Col5 \right)$, see Fig S2G  $\int_{0 V}^{0.875 V} f\left( V \right)\approx\left( L3, Col5 \right)+\frac{5\Delta V}{288}\left( 19f\left( 0.875 V \right)+75f\left( 0.75 V \right)+50f\left( 0.625 V \right)+50f\left( 0.5 V \right)+75f\left( 0.375 V \right)+19f\left( 0.25 V \right) \right)=0.03421 mW+\frac{5\left( 0.125 V \right)}{288}\left( 19\times121.34 mA+75\times51.7629 mA+50\times13.8466 mA+50\times2.7596 mA+75\times0.7097 mA+19\times0.2974 mA \right)=15.392 mW$ | $15.392 mW$ |
| L9 | 1 | 209.615 | 210.614 | Order 5 Simpson integration formula is used in this case, adding it to the result in $\left( L4, Col5 \right)$, see Fig S2H  $\int_{0 V}^{1 V} f\left( V \right)\approx\left( L4, Col5 \right)+\frac{5\Delta V}{288}\left( 19f\left( 1 V \right)+75f\left( 0.875 V \right)+50f\left( 0.75 V \right)+50f\left( 0.625 V \right)+75f\left( 0.5 V \right)+19f\left( 0.375 V \right) \right)=0.09349 mW+\frac{5\left( 0.125 V \right)}{288}\left( 19\times210.614 mA+75\times121.34 mA+50\times51.7629 mA+50\times13.8466 mA+75\times2.7596 mA+19\times0.7097 mA \right)=36.1246 mW$ | $36.1246 mW$ |
