# Supplementary material for: Five solar cell parameters automatic extraction, within the one diode-solar cell model, using the implemented Simpson order 5 integration method, in an executable program
Source: PLoS One. 2026 Apr 22;21(4):e0346051. doi: 10.1371/journal.pone.0346051 (PMC13102238; doi:10.1371/journal.pone.0346051)
Supplement: S2 Table — The value “nan” means “not-a-number”, and it appears in N=6 case, as the standard deviation is undetermined, as explained in the text (see Eq. (35)). (DOCX) [file pone.0346051.s006.docx]

**S2 Table**. $C_{V0} \pm{\Delta C}_{V0},$ $C_{V1}\pm{\Delta C}_{V1}, {C_{V2}\pm{\Delta C}_{V2},C}_{I1}\pm{\Delta C}_{I1}, C_{I2}\pm\Delta C_{I2}$*,* $C_{V1I1}\pm{\Delta C}_{V1I1}, R_{s}\pm\Delta R_{s}$*,* $R_{sh}\pm\Delta R_{sh}, n\pm\Delta n, I_{lig}\pm\Delta I_{lig}, \mathrm{and} I_{sat}\pm\Delta I_{sat}$ obtained using the program CCSimpsonOrder5.exe. The value “nan” means “not-a-number”, and it appears in
$N=6$ case, as the standard deviation is undetermined, as explained in the text (see Eq. (35)).

| $C_{V0} \pm{\Delta C}_{V0},$ $C_{V1}\pm{\Delta C}_{V1}, {C_{V2}\pm{\Delta C}_{V2},C}_{I1}\pm{\Delta C}_{I1}, C_{I2}\pm\Delta C_{I2}$*,* and $C_{V1I1}\pm{\Delta C}_{V1I1}$ | | | | | | |
| --- | --- | --- | --- | --- | --- | --- |
| $N$ | $C_{V0} \pm{\Delta C}_{V0}$ | $C_{V1}\pm\Delta C_{V1}$ | $C_{V2}\pm\Delta C_{V2}$ | $C_{I1}\pm\Delta C_{I1}$ | $C_{I2}\pm\Delta C_{I2}$ | $C_{V1I1}\pm{\Delta C}_{V1I1}$ |
| 6 | -2.33299e-016 ± nan | -0.000206906 ± nan | 0.000424311 ± nan | 0.271645 ± nan | 0.982413 ± nan | -0.307694 ± nan |
| 7 | 2.34748e-007 ± 1.27354e-006 | -0.000134572 ± 1.28898e-005 | 0.000582316 ± 3.12309e-005 | 0.129506 ± 0.00216099 | 0.675083 ± 0.003902 | -0.101549 ± 0.00289438 |
| 8 | 1.29233e-006 ± 4.14978e-006 | -0.000114062 ± 4.00318e-005 | 0.000688908 ± 9.13193e-005 | 0.0575262 ± 0.00572977 | 0.504299 ± 0.0102677 | 0.00519762 ± 0.00764541 |
| 9 | 4.62392e-006 ± 1.66578e-005 | -0.000169591 ± 0.000157949 | 0.000920198 ± 0.00035395 | 0.0178221 ± 0.0215788 | 0.416255 ± 0.0385621 | 0.0621367 ± 0.0287502 |
| 10 | 3.54664e-006 ± 1.12189e-005 | -0.000136319 ± 0.000105056 | 0.000785064 ± 0.000232217 | 0.0304424 ± 0.0138314 | 0.43611 ± 0.0246791 | 0.045667 ± 0.018409 |
| 11 | 1.81604e-006 ± 3.69025e-006 | -9.86538e-005 ± 3.42037e-005 | 0.000640022 ± 7.48032e-005 | 0.04611 ± 0.00438645 | 0.464682 ± 0.00782479 | 0.0244616 ± 0.00583539 |
| 19 | -9.89992e-008 ± 2.22197e-007 | -6.39231e-005 ± 1.98219e-006 | 0.00049526 ± 4.18091e-006 | 0.064957 ± 0.000235089 | 0.501239 ± 0.000421515 | -0.00152944 ± 0.000312765 |
| 26 | -3.53897e-009 ± 1.51521e-008 | -6.54472e-005 ± 1.33399e-007 | 0.000499843 ± 2.782e-007 | 0.0645679 ± 1.55021e-005 | 0.5005 ± 2.79074e-005 | -0.0010019 ± 2.06386e-005 |
| 38 | -8.78605e-010 ± 1.56975e-009 | -6.55012e-005 ± 1.36682e-008 | 0.000499958 ± 2.82484e-008 | 0.064569 ± 1.56539e-006 | 0.500507 ± 2.83016e-006 | -0.00100478 ± 2.08583e-006 |
| 51 | -4.17962e-010 ± 1.94184e-009 | -6.55095e-005 ± 1.68068e-008 | 0.000499981 ± 3.45702e-008 | 0.0645676 ± 1.91142e-006 | 0.500504 ± 3.46509e-006 | -0.00100289 ± 2.54834e-006 |
| 64 | -3.58354e-010 ± 1.51026e-009 | -6.55107e-005 ± 1.30251e-008 | 0.000499985 ± 2.67183e-008 | 0.0645671 ± 1.47571e-006 | 0.500503 ± 2.67979e-006 | -0.00100215 ± 1.96816e-006 |
| 76 | -3.50832e-010 ± 6.96514e-010 | -6.55112e-005 ± 5.99387e-009 | 0.000499985 ± 1.22747e-008 | 0.0645675 ± 6.77581e-007 | 0.500504 ± 1.23179e-006 | -0.00100283 ± 9.03906e-007 |
| 101 | -5.35137e-010 ± 1.19855e-009 | -6.55089e-005 ± 1.02845e-008 | 0.000499979 ± 2.10156e-008 | 0.0645678 ± 1.15937e-006 | 0.500505 ± 2.11081e-006 | -0.00100318 ± 1.54713e-006 |
| 251 | -2.0776e-010 ± 7.08676e-010 | -6.55132e-005 ± 6.04938e-009 | 0.000499992 ± 1.2314e-008 | 0.0645665 ± 6.78789e-007 | 0.500502 ± 1.23939e-006 | -0.00100138 ± 9.06386e-007 |
| 501 | -9.29654e-011 ± 4.76848e-010 | -6.55145e-005 ± 4.06337e-009 | 0.000499996 ± 8.26085e-009 | 0.0645659 ± 4.55288e-007 | 0.500501 ± 8.32139e-007 | -0.00100055 ± 6.08081e-007 |
| 751 | 5.09865e-012 ± 4.00148e-010 | -6.55157e-005 ± 3.4078e-009 | 0.0005 ± 6.92517e-009 | 0.0645656 ± 3.81656e-007 | 0.5005 ± 6.97798e-007 | -0.00100008 ± 5.09777e-007 |
| 1001 | -1.53769e-010 ± 3.18157e-010 | -6.55138e-005 ± 2.70874e-009 | 0.000499994 ± 5.50342e-009 | 0.0645662 ± 3.03295e-007 | 0.500501 ± 5.54623e-007 | -0.00100096 ± 4.05127e-007 |
| 2501 | -7.20198e-011 ± 2.09737e-010 | -6.55148e-005 ± 1.78474e-009 | 0.000499997 ± 3.62473e-009 | 0.0645658 ± 1.99753e-007 | 0.500501 ± 3.65393e-007 | -0.00100046 ± 2.66839e-007 |
| 5001 | -3.63061e-011 ± 1.49476e-010 | -6.55152e-005 ± 1.27173e-009 | 0.000499999 ± 2.58251e-009 | 0.0645657 ± 1.42317e-007 | 0.5005 ± 2.60356e-007 | -0.00100022 ± 1.90117e-007 |
| 7501 | 2.12024e-011 ± 1.20038e-010 | -6.55159e-005 ± 1.02121e-009 | 0.000500001 ± 2.07369e-009 | 0.0645654 ± 1.14276e-007 | 0.5005 ± 2.09066e-007 | -0.000999902 ± 1.5266e-007 |
| 10001 | 1.7193e-011 ± 1.06116e-010 | -6.55158e-005 ± 9.02751e-010 | 0.000500001 ± 1.83311e-009 | 0.0645654 ± 1.01018e-007 | 0.5005 ± 1.84813e-007 | -0.000999889 ± 1.34949e-007 |
| 12501 | 2.97307e-011 ± 9.44102e-011 | -6.5516e-005 ± 8.03149e-010 | 0.000500001 ± 1.63084e-009 | 0.0645654 ± 8.98714e-008 | 0.5005 ± 1.64422e-007 | -0.000999821 ± 1.20058e-007 |
| 15001 | 2.48106e-011 ± 8.58084e-011 | -6.55159e-005 ± 7.29965e-010 | 0.000500001 ± 1.48222e-009 | 0.0645654 ± 8.16814e-008 | 0.5005 ± 1.49439e-007 | -0.000999825 ± 1.09118e-007 |
| 17501 | -4.17708e-011 ± 8.05971e-011 | -6.55151e-005 ± 6.85627e-010 | 0.000499998 ± 1.39218e-009 | 0.0645657 ± 7.67197e-008 | 0.5005 ± 1.40362e-007 | -0.00100024 ± 1.02489e-007 |
| 20001 | 2.36059e-011 ± 7.53082e-011 | -6.55159e-005 ± 6.40632e-010 | 0.000500001 ± 1.30081e-009 | 0.0645654 ± 7.16844e-008 | 0.5005 ± 1.31151e-007 | -0.000999877 ± 9.5763e-008 |
| Solar cell parameters obtained using the program, and the previous values of $C_{V0} \pm{\Delta C}_{V0},$ $C_{V1}\pm{\Delta C}_{V1}, {C_{V2}\pm{\Delta C}_{V2},C}_{I1}\pm{\Delta C}_{I1}, C_{I2}\pm\Delta C_{I2}$*, and* $C_{V1I1}\pm{\Delta C}_{V1I1}$ | | | | | | |
| $N$ | $R_{s}\pm\Delta R_{s}$ $(Ω$) | $R_{sh}\pm\Delta R_{sh} (Ω$) | $n\pm\Delta n$ | $I_{lig}\pm\Delta I_{lig} (A)$ | $I_{sat}\pm\Delta I_{sat} (A)$ | |
| 6 | 1.96156 ± nan | 1178.38 ± nan | 10.5131 ± nan | 0.000977375 ± nan | 0.0268011 ± nan | |
| 7 | 1.34805 ± 0.0153329 | 858.64 ± 46.0508 | 5.01257 ± 0.0843922 | 0.00098451 ± 2.34105e-005 | 0.000812972 ± 4.17692e-005 | |
| 8 | 1.0072 ± 0.0405875 | 725.786 ± 96.2076 | 2.22525 ± 0.223556 | 0.00103533 ± 5.82526e-005 | 2.23597e-007 ± 1.08041e-007 | |
| 9 | 0.831238 ± 0.152803 | 543.362 ± 209.001 | 0.685313 ± 0.84097 | 0.00113756 ± 0.000209668 | 1.079e-021 ± 1.49715e-020 | |
| 10 | 0.871028 ± 0.0977444 | 636.89 ± 188.388 | 1.17534 ± 0.539386 | 0.00108906 ± 0.000140509 | 4.06841e-013 ± 1.36871e-012 | |
| 11 | 0.92826 ± 0.0309677 | 781.223 ± 91.3063 | 1.78366 ± 0.171188 | 0.00103992 ± 4.65833e-005 | 5.20003e-009 ± 2.70202e-009 | |
| 19 | 1.00148 ± 0.00166598 | 1009.57 ± 8.52263 | 2.51523 ± 0.00918683 | 0.000999622 ± 2.75039e-006 | 1.07193e-006 ± 1.69766e-008 | |
| 26 | 0.999999 ± 0.000110296 | 1000.32 ± 0.556751 | 2.50009 ± 0.000605886 | 0.00100095 ± 1.84304e-007 | 9.90915e-007 ± 1.14664e-009 | |
| 38 | 1.00001 ± 1.11853e-005 | 1000.08 ± 0.0565062 | 2.50014 ± 6.11893e-005 | 0.00100099 ± 1.8829e-008 | 9.91171e-007 ± 2.12492e-010 | |
| 51 | 1.00001 ± 1.36947e-005 | 1000.04 ± 0.0691459 | 2.50008 ± 7.472e-005 | 0.00100099 ± 2.31183e-008 | 9.90883e-007 ± 2.35751e-010 | |
| 64 | 1.00001 ± 1.05911e-005 | 1000.03 ± 0.0534398 | 2.50006 ± 5.76894e-005 | 0.001001 ± 1.79015e-008 | 9.90781e-007 ± 2.06512e-010 | |
| 76 | 1.00001 ± 4.86833e-006 | 1000.03 ± 0.0245509 | 2.50008 ± 2.64889e-005 | 0.001001 ± 8.23379e-009 | 9.90863e-007 ± 1.52914e-010 | |
| 101 | 1.00001 ± 8.3425e-006 | 1000.04 ± 0.0420348 | 2.50009 ± 4.53248e-005 | 0.00100099 ± 1.41188e-008 | 9.90925e-007 ± 1.85335e-010 | |
| 251 | 1 ± 4.8985e-006 | 1000.02 ± 0.0246288 | 2.50004 ± 2.65378e-005 | 0.001001 ± 8.29553e-009 | 9.90661e-007 ± 1.53017e-010 | |
| 501 | 1 ± 3.28893e-006 | 1000.01 ± 0.0165219 | 2.50002 ± 1.78001e-005 | 0.001001 ± 5.57012e-009 | 9.90542e-007 ± 1.37975e-010 | |
| 751 | 1 ± 2.75797e-006 | 1000 ± 0.0138503 | 2.5 ± 1.49214e-005 | 0.001001 ± 4.67091e-009 | 9.90469e-007 ± 1.33014e-010 | |
| 1001 | 1 ± 2.19209e-006 | 1000.01 ± 0.0110071 | 2.50003 ± 1.18578e-005 | 0.001001 ± 3.71253e-009 | 9.906e-007 ± 1.27758e-010 | |
| 2501 | 1 ± 1.44419e-006 | 1000.01 ± 0.00724954 | 2.50001 ± 7.8097e-006 | 0.001001 ± 2.44586e-009 | 9.90529e-007 ± 1.20783e-010 | |
| 5001 | 1 ± 1.02904e-006 | 1000 ± 0.00516506 | 2.50001 ± 5.56413e-006 | 0.001001 ± 1.74276e-009 | 9.90494e-007 ± 1.16914e-010 | |
| 7501 | 0.999999 ± 8.26317e-007 | 999.999 ± 0.00414738 | 2.5 ± 4.46784e-006 | 0.001001 ± 1.39943e-009 | 9.90444e-007 ± 1.15022e-010 | |
| 10001 | 1 ± 7.3046e-007 | 999.999 ± 0.0036662 | 2.5 ± 3.94948e-006 | 0.001001 ± 1.23709e-009 | 9.90447e-007 ± 1.1413e-010 | |
| 12501 | 0.999999 ± 6.49866e-007 | 999.998 ± 0.00326166 | 2.5 ± 3.51368e-006 | 0.001001 ± 1.1006e-009 | 9.90437e-007 ± 1.13379e-010 | |
| 15001 | 1 ± 5.90648e-007 | 999.998 ± 0.00296443 | 2.5 ± 3.19348e-006 | 0.001001 ± 1.00031e-009 | 9.90438e-007 ± 1.12828e-010 | |
| 17501 | 1 ± 5.54772e-007 | 1000 ± 0.00278438 | 2.50001 ± 2.99949e-006 | 0.001001 ± 9.39547e-010 | 9.90498e-007 ± 1.125e-010 | |
| 20001 | 1 ± 5.18363e-007 | 999.998 ± 0.00260162 | 2.5 ± 2.80263e-006 | 0.001001 ± 8.77887e-010 | 9.90445e-007 ± 1.12156e-010 | |
